# Comprehensive comparison of gene expression diversity among a variety of human stem cells

**DOI:** 10.1093/nargab/lqac087

**Published:** 2022-11-29

**Authors:** Yukiyo Yamatani, Kenta Nakai

**Affiliations:** Department of Computational Biology and Medical Sciences, the University of Tokyo, 5-1-5 Kashiwanoha, Kashiwa-shi, Chiba 277-8562, Japan; Department of Computational Biology and Medical Sciences, the University of Tokyo, 5-1-5 Kashiwanoha, Kashiwa-shi, Chiba 277-8562, Japan; Human Genome Center, the Institute of Medical Science, the University of Tokyo, 4-6-1 Shirokanedai Minato-ku, Tokyo 108-8639, Japan

## Abstract

Several factors, including tissue origins and culture conditions, affect the gene expression of undifferentiated stem cells. However, understanding the basic identity across different stem cells has not been pursued well despite its importance in stem cell biology. Thus, we aimed to rank the relative importance of multiple factors to gene expression profile among undifferentiated human stem cells by analyzing publicly available RNA-seq datasets. We first conducted batch effect correction to avoid undefined variance in the dataset as possible. Then, we highlighted the relative impact of biological and technical factors among undifferentiated stem cell types: a more influence on tissue origins in induced pluripotent stem cells than in other stem cell types; a stronger impact of culture condition in embryonic stem cells and somatic stem cell types, including mesenchymal stem cells and hematopoietic stem cells. In addition, we found that a characteristic gene module, enriched in histones, exhibits higher expression across different stem cell types that were annotated by specific culture conditions. This tendency was also observed in mouse stem cell RNA-seq data. Our findings would help to obtain general insights into stem cell quality, such as the balance of differentiation potentials that undifferentiated stem cells possess.

## INTRODUCTION

Stem cells are important sources for regenerative therapy and drug discovery. There are two major classes of stem cells: pluripotent stem cells (1) that possess the ability to differentiate into all three germ layers (2–5) and somatic stem cells (6) that can differentiate into multiple lineages within their tissue origins (7–9). The former includes embryonic stem cells (ESCs) and induced pluripotent stem cells (iPSCs) while the latter includes mesenchymal stem cells (MSCs) and hematopoietic stem cells (HSCs).

Phenotypes and differentiation potentials of stem cells cultured *in vitro* are determined by their gene expression profiles (10–12). Several factors, including tissue origins (biological ones) and culture conditions (technical ones), affect their gene expression profiles, sometimes resulting in changing their state or identity (13–16).

Many previous studies have reported the impact of factors on pluripotent and somatic stem cell types, in which findings on their differential potential into tissue lineage were shown (17–23). In pluripotent stem cells, iPSCs derived from the endothelial cells of the umbilical cord vein exhibited higher differentiation potential toward endothelial or hematopoietic lineage compared to dermal fibroblast-derived ones (17). In iPSCs cultured in relatively shorter terms (5–9 passages), it has been reported that the degree of passages can affect both the epigenetic memory of iPSCs (18) and the similarity between ESCs and iPSCs (19). In somatic stem cells, characteristic gene expression patterns among perinatal tissue-derived MSCs or hematopoietic tissue-derived HSCs were observed (20,21). A significant impact of passages on MSCs has also been reported. Bone marrow-derived MSCs (>3 passages) increased the cytokine secretome (22) and adipose-derived MSCs cultured in different passages (3, 6, 9 and 12 passages) exhibited differential expression patterns of secreted proteins (23).

Most of these studies have identified just the existence of the impact of the factors on the differential potential of both pluripotent and somatic stem cells. However, these studies were not systematic across various stem cell types. Due to a lack of basic knowledge, observations were sometimes controversial. For example, on the effect of different compositions in the cell culture medium of pluripotent stem cells, opposite findings were reported: a group reported that xeno-free culture medium, which is free from non-human derived ingredients, did not maintain the stemness and proliferation (24), whereas another group reported that the xeno-free medium enhanced the stability of undifferentiated stem cells (25). Between adipose- and bone marrow-derived MSCs, an increased adipogenic potential in adipose-derived MSCs, and vice versa in the osteogenic potential in bone marrow-derived MSCs were observed (26), to which some researchers reported opposite findings (27,28). Thus, the extent to which biological and technical factors affect gene expression diversity encompassing a wider variety of undifferentiated stem cells has been lacking despite the importance of understanding stem cell identity.

To fill this gap, we relatively characterized the susceptibility of the factors, such as tissue origins and culture conditions, on the gene expression profiles of various undifferentiated human stem cells. We analyzed public RNA sequencing (RNA-seq) data of four stem cell types (ESCs, iPSCs, MSCs and HSCs) generated by multiple research groups and annotated with these factors. To avoid undefined factors (batch effects) which can affect the gene expression profiles, including experimental biases depending on the laboratories where the experiments were performed (29,30), we conducted batch effect correction, followed by a dimensionality reduction, stem cell clustering, and correlation analysis to identify factors and genes that are characteristic for stem cell clusters. Through the analysis, we identified the relative impact of biological and technical factors among the stem cell types, suggesting a new insight into stem cell features.

## MATERIALS AND METHODS

### Data preparation

103 (bulk) RNA-seq data of undifferentiated human stem cells reported by 24 different research groups (21,31–47) were downloaded from the NCBI Gene Expression Omnibus (GEO). All data were derived from healthy donor cells without any differentiation induction. They were distinguished into four types (ESCs, iPSCs, MSCs and HSCs). Research groups were identified with their GEO series accession IDs (GSE IDs). The identifiers were listed in Table 1. Downloaded data were annotated with biological factors such as tissue origins and technical factors, including passages and culture medium composition, based on the information obtained using the GEO accession viewer. In all stem cell types except ESCs that were commonly derived from inner cell mass, their tissue origins were as follows: skin fibroblast, amniotic membrane and peripheral blood for iPSCs; adipose, bone marrow, umbilical cord, amniotic membrane and chorionic plates of placenta for MSCs; umbilical cord, bone marrow and fetal liver for HSCs. The annotated passages were classified into three terms as follows: the short-passage term (1–2 passages), the middle-passage term (3–7 passages) and the long-passage term (>10 passages). Characteristics related to culture medium compositions, such as xenogenic ingredient, L-glutamine (L-GLN), serum or growth factor (SCF: stem cell factor; FLT3: FMS-related tyrosine kinase 3; and TPO: Thrombopoietin) were obtained. The detailed data information are listed in Supplementary Table S1(A–E). Additional microarray expression profiles of undifferentiated human iPSCs annotated with their passages (10–67 passages) (GSE42445) (48) and RNA-seq data of undifferentiated mouse stem cells (49–53) were also downloaded from GEO. The mouse dataset (Supplementary Table S9) was used for histone gene expression analysis.

**Table 1.** Downloaded RNA-seq data description including the research group ID (Nos. 1–24), the GSE IDs of the NCBI GEO database, the stem cell types, and the number of data provided for each group

Research group ID	GSE ID	Stem cell type	#Data	Reference
No.1	GSE148158	ESC	2	(31)
No.2	GSE138170		2	(32)
No.3	GSE154481		3	Unknown
No.4	GSE127201		2	(33)
No.5	GSE159926	iPSC	3	(34)
No.6	GSE147498		2	(35)
No.7	GSE116471		2	(36)
No.8	GSE65423		3	Unknown
No.9	GSE113253	MSC	7	(37)
No.10	GSE125331		2	(38)
No.11	GSE30567		5	(39)
No.12	GSE60237		2	(40)
No.13	GSE96788		3	(41)
No.14	GSE85223		3	(42)
No.15	GSE137186		18	Unknown
No.16	GSE115240		4	(43)
No.17	GSE90274		2	(44)
No.18	GSE131355		4	Unknown
No.19	GSE118808		11	(45)
No.20	GSE107149	HSC	4	Unknown
No.21	GSE109093		9	(21)
No.22	GSE110968		3	Unknown
No.23	GSE69932		2	(46)
No.24	GSE63569		5	(47)

### RNA-seq data preprocessing

First, we performed a quality check and adapter sequence detection using FastQC (https://www.bioinformatics.babraham.ac.uk/projects/fastqc/). Then, we performed Trimmomatic (54) to trim reads with low-quality scores (<20) and short base lengths (<50) as well as to remove detected adapter sequences. Second, we mapped the remaining reads into the reference genome (hg38 for humans) by HISAT2 (55). 19299 genes remained after the read mapping. Third, we filtered against duplicated reads, repeat sequences and the reads mapped to ribosomal RNAs. Then, we quantified read counts by featureCounts (56) and removed ones with less than three counts in all samples. After the filtering, 15 918 genes remained. Finally, we normalized the filtered read counts into transcripts per million (TPM).

### Batch effect correction and stemness confirmation

We performed RUVSeq (57) to correct batch effects in the gene expression data. Namely, unwanted variations (e.g. experimental biases depending on the laboratories) that could be significant batch effects were estimated by RUVg, one of the functions in RUVSeq, using housekeeping gene sets (58) as negative controls. The list of 3804 housekeeping genes was downloaded from http://www.tau.ac.il/∼elieis/HKG/. We conducted the batch effect correction for 9679 genes in total. To evaluate the effect of the correction, we measured the ratio of the variance of gene expression profile between different stem cell samples/variance within the same stem cell samples, in terms of four kinds of stem cell types or 24 kinds of research groups. Principal component analysis (PCA) was used for visualizing the variation of gene expression profile among stem cell samples. Moreover, we investigated the expression levels of stem cell type-related marker genes (21,59–64) and differentiation marker genes (65–68) to confirm the stemness of cells whose RNAs were used for generating RNA-seq data. The maker gene information is shown in Supplementary Tables S2 and S3.

### Dimensionality reduction and clustering

To do cluster analysis and visualize multiple stem cells with their similarity in two dimensions, we performed dimensionality reduction with the uniform manifold approximation and projection (UMAP) using the Seurat package following the approach for bulk RNA-seq data by Yang *et al.* (69,70). Gene expression data after batch-effect correction were used. Prior to conducting UMAP, PCA was performed (*npcs* = 30) as an initial dimensionality reduction of the input data, following filtering out genes with low variance (lower than the coefficient of variation of 0.8 among all samples) to screen the most characteristically expressed genes. The number of significant principal components used as the dimension parameter in UMAP was determined using the JackStraw function. Then, UMAP and unsupervised clustering were performed (*dimension* = 5, *n.neighbors* = 10), followed by clustering using a K-nearest neighbor (KNN) graph, (*resolution* = 1.1). Stem cell clusters obtained from the UMAP and KNN graph were assigned into clusters and subclusters based on their gene expression profile. The stability of the clustering result was confirmed by conducting t-distributed stochastic neighbor embedding (t-SNE) as another dimensionality reduction method (71) with the same parameters used in the UMAP clustering and hierarchical clustering with Ward’s method. We have explained the parameter choice of UMAP and clustering in the supplementary file (Supplementary Method S1 and Supplementary Figure S1).

### Evaluating the factor impact in each stem cell type

To investigate the extent to which biological and technical factors impact gene expression profiles among stem cells, we evaluated the impact of tissue origins in each stem cell type by investigating the expression pattern of the genes characteristic of skin fibroblast (72–74), amniotic membrane (75–77), adipose (26), bone marrow (78,79), umbilical cord (80) and peripheral blood (81). The list of genes is shown in Supplementary Table S4. In addition, interrelations of all annotations including cluster classification, tissue origins and technical factors in each stem cell type were investigated. Using the swamp package (https://CRAN.R-project.org/package = swamp), each interrelation was applied to a linear model and then, the significance of the model was tested using the chi-square test.

### Differential gene expression analysis

We detected differentially expressed genes (DEGs) from stem cells with different factor annotations. We used DESeq2 (82) by setting false discovery rate (FDR) < 0.05 and |FC: Fold change| ≥ 2 as cutoff threshold. Then, we conducted the gene functional enrichment analysis using Gene Ontology (http://www.geneontology.org). We detected significantly enriched gene ontology (GO) terms (*P*-value < 0.01 and fold enrichment ≥ 2) from the DEGs. We extracted the top 10 enrichment results with the highest fold enrichment ratio to identify representative gene functions.

### Weighted gene co-expression network analysis

We constructed co-expression networks through WGCNA (83) and identified gene modules that were highly correlated with each stem cell cluster. The same input data used in UMAP and the stem cell cluster annotation were used. First, a co-expression network was constructed to determine the connection strengths between two genes by using an appropriate soft thresholding power (14, scale-free *R*^2^ = 0.88), selected based on scale-free fitting. Second, Topological Overlap Matrix (TOM) matrix, which is the relationship between gene pair expression, is defined to reflect the similarity between genes at the expression and the network topology level. Then, another adjacency matrix that considers topological similarity, the dissimilarity matrix (1-TOM) obtained from TOM, was built. Third, hierarchical clustering of genes was performed to identify key modules (with the minimal module size of 100 and sensitive module detection parameter 3). The obtained gene modules with high similarity were merged (with the height cut-off of 0.15). Third, to screen and prioritize potential genes that are strongly characteristic of the clusters, the correlation between the module eigengene, which is defined as the first principal component of gene expressions in each module (84), and the stem cell clusters was measured with Pearson's correlation coefficient (PCC). The gene significance (GS), which is defined as the association with each gene expression level and the clusters, as well as the module membership (MM), which is defined as the association with the gene expression profile and the module eigengene, were used to identify potential genes significantly correlated with stem cell clusters (GS > 0.5 and MM > 0.8). We explained the parameter choice of WGCNA in the supplementary file (Supplementary Method S2, Supplementary Figure S4, and Supplementary Tables S6 and S7). Finally, we extracted the top 10 enrichment results with the highest fold enrichment ratio (*P*-value < 0.01 and fold enrichment ≥ 2) in each module to identify representative gene functions. Moreover, to investigate genes that exhibit somewhat remarkable expression patterns of the modules, we compared the expression levels of module genes that corresponded with the DEGs of each stem cell among the clusters. Statistical significance was determined using Tukey's multiple comparison test with ANOVA (adjusted *P*-value < 0.01).

## RESULTS

### Batch effect correction and stemness confirmation

**Figure 1.**
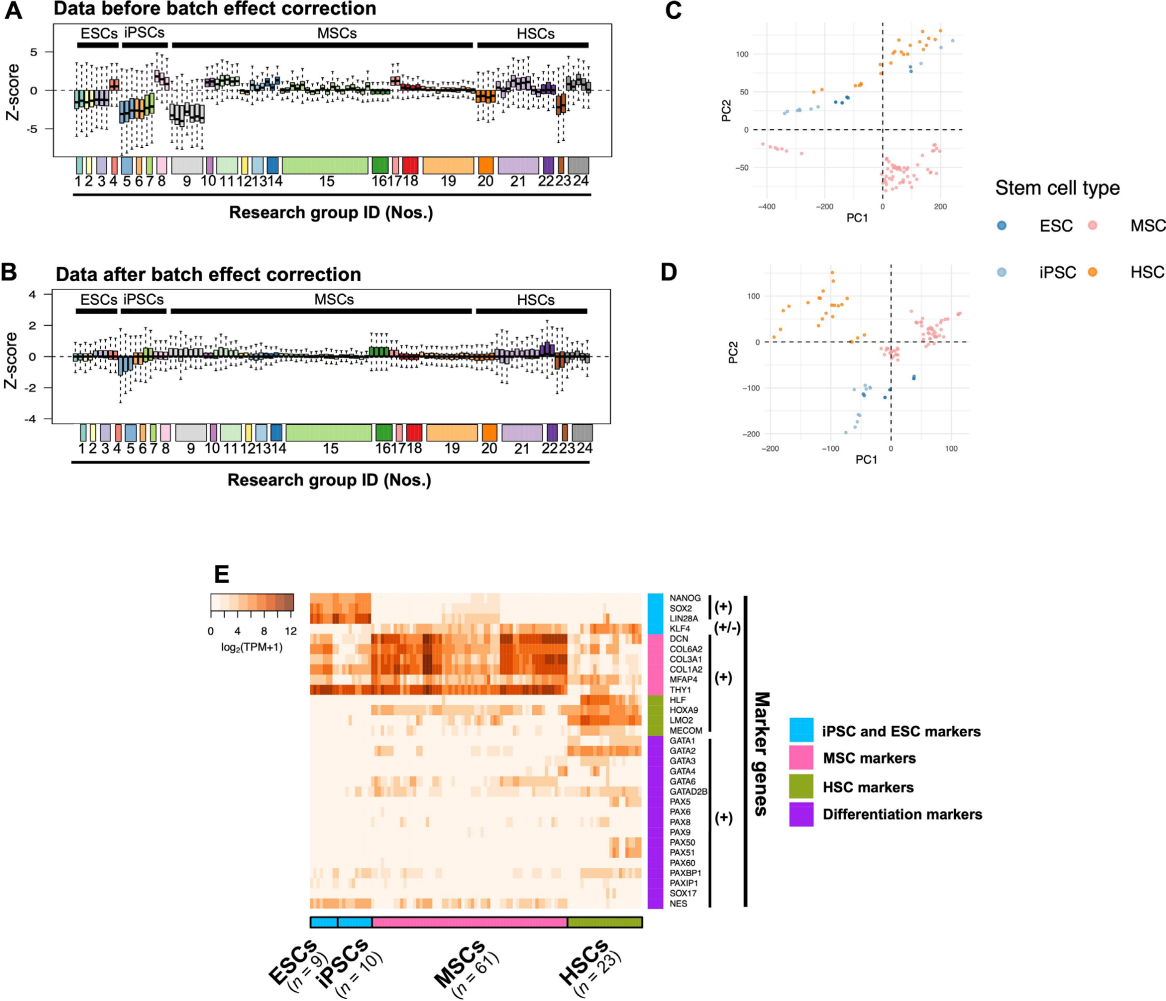
Batch effect correction and stemness confirmation in the stem cell gene expression data. (**A** and **B**) Relative gene expression levels (in *Z*-score) among the RNA-seq data before and after the batch effect correction, respectively. The numbers along the horizontal axis represent research groups listed in Supplementary Table S1. Different colors were used for their easy distinction. (**C** and **D**) PCA of RNA-seq data (C) before and (D) after the batch effect correction, respectively. The colors of the dots represent the stem cell types, as shown in the figure. (**E**) Heatmap showing the log2-fold transcripts per million (TPM) of the stem cell-related marker genes for ESCs/iPSCs (sky blue), MSCs (magenta), and HSCs (green) and differentiation markers (purple). The darker orange color represents higher gene expression. Expression of positive (+) and negative (-) marker genes are shown, where the color bar on the *y*-axis shows the distinction of the stem cell types and the color bar on the *x*-axis also shows the corresponding cell types for each marker gene.

We first conducted batch effect correction for 9679 gene expression data, based on the correction of estimated batch effects among housekeeping genes. We compared the relative expression levels of genes before and after the batch effect correction. The deviation of expression levels was larger visualized depending on research groups than stem cell types before the correction (Figure 1A), while it was smaller after the correction (Figure 1B), which suggested that significant batch effects were reduced, and the gene expression profile reflected more biological features based on the stem cell types. In addition, we measured the ratio of the variance of gene expression profile among stem cell types or research groups, and compared it, respectively. After the correction, we observed that the ratio of variance among the stem cell types was larger (13.3 before the correction and 17.3 after the correction), whereas that among research groups was smaller (41.4 before the correction and 27.6 after the correction), implying that the deviation by the research groups that could be batch effects was reduced and the biological differences among stem cell types became more apparent. The distinct expression profile depending on stem cell types was confirmed by separative distribution after the correction (Figure 1C and D).

Next, we investigated the expression patterns of 14 marker genes for the stem cell types which have been previously reported (Figure 1E). In pluripotent stem cells (ESCs and iPSCs), their positive markers (NANOG, SOX2, and LIN28A) exhibited remarkably high expression specific to both types of cells, whereas KLF4 exhibited lower expression, which corresponds with previous findings (62,63). All six positive markers for mesenchymal lineage cells (DCN, COL6A2, COL3A1, COL1A2, MFAP4 and THY1) were highly expressed in MSCs. Positive markers for hematopoietic stem cells (HLF, HOXA9, LMO2 and MECOM) showed higher expression compared to the other stem cell types. Thus, we indicated that overall these cells show undifferentiated stem cell identity. We also confirmed expression patterns of 17 positive differentiation markers related to GATAs, PAXs, SOX17 and NES. Most of the differentiated markers exhibited relatively lower expression patterns than the stem cell marker genes in each stem cell type.

### Dimensionality reduction using UMAP and clustering of stem cells

**Figure 2.**
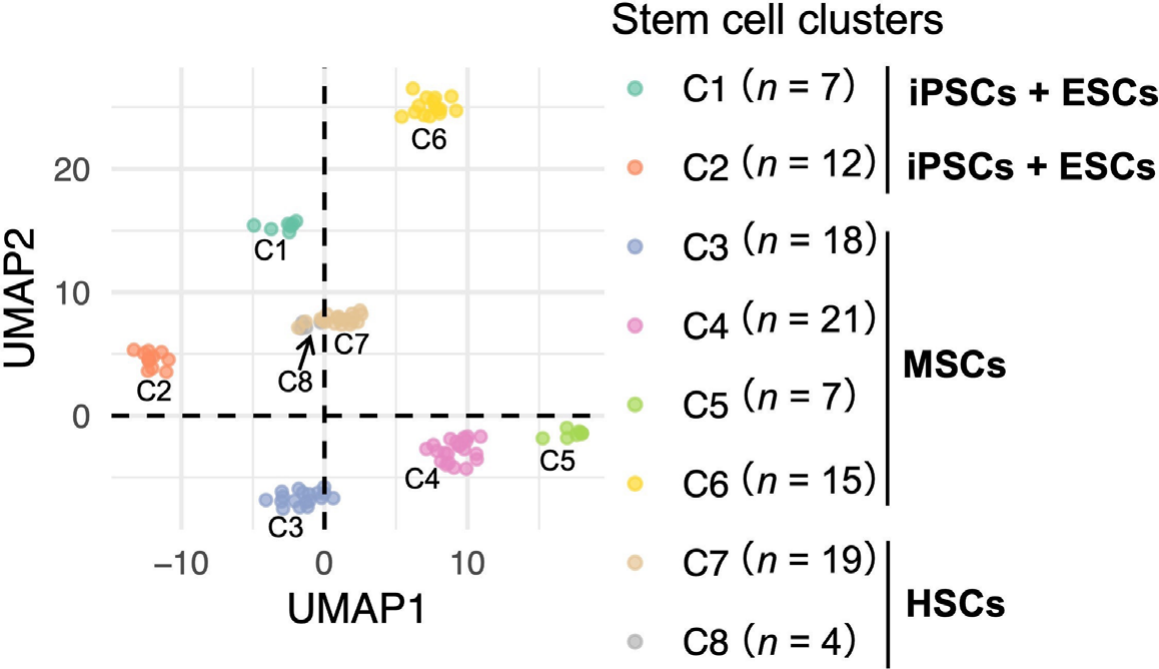
Stem cell clustering. The stem cell cluster distribution was obtained using a K-nearest neighbor (KNN) graph with the relative similarity of gene expression profiles among stem cells, following dimensionality reduction by UMAP. The individual dot represents each stem cell sample. The colors of the dots are used to distinguish the stem cell clusters (C1–C8). The black arrow points C8.

To capture the global similarity of the gene expression profiles among stem cells, we conducted dimensionality reduction by UMAP using the batch effect-corrected 9679 gene expression data among stem cells that belong to the four types (ESCs, iPSCs, MSCs and HSCs). We classified all stem cells into eight clusters (C1–C8) based on the clustering using a KNN graph with the relative similarity of gene expression profiles among stem cells (Figure 2 and Supplementary Figure S1B). The stability of the result was confirmed by conducting another dimensionality reduction method, t-SNE (Supplementary Figure S1C), and hierarchical clustering with Ward’s method (Supplementary Figure S1D). Both C1 (*n* = 7) and C2 (*n* = 12) included ESCs and iPSCs. MSCs were divided into four clusters as follows: C3 (*n* = 18), C4 (*n* = 21), C5 (*n* = 7) and C6 (*n* = 15). HSCs were divided into C7 (*n* = 19) and C8 (*n* = 4). As expected, pluripotent stem cell clusters (C1 and C2) and somatic stem cell clusters (C3–C8) were separated, coinciding with previous reports on their remarkable differences in the proliferation time and the differentiation potential (85,86). The correspondence between individual stem cells among replicates was also confirmed (Supplementary Table S1).

### Comparison of tissue-origin effects across stem cell types

**Figure 3.**
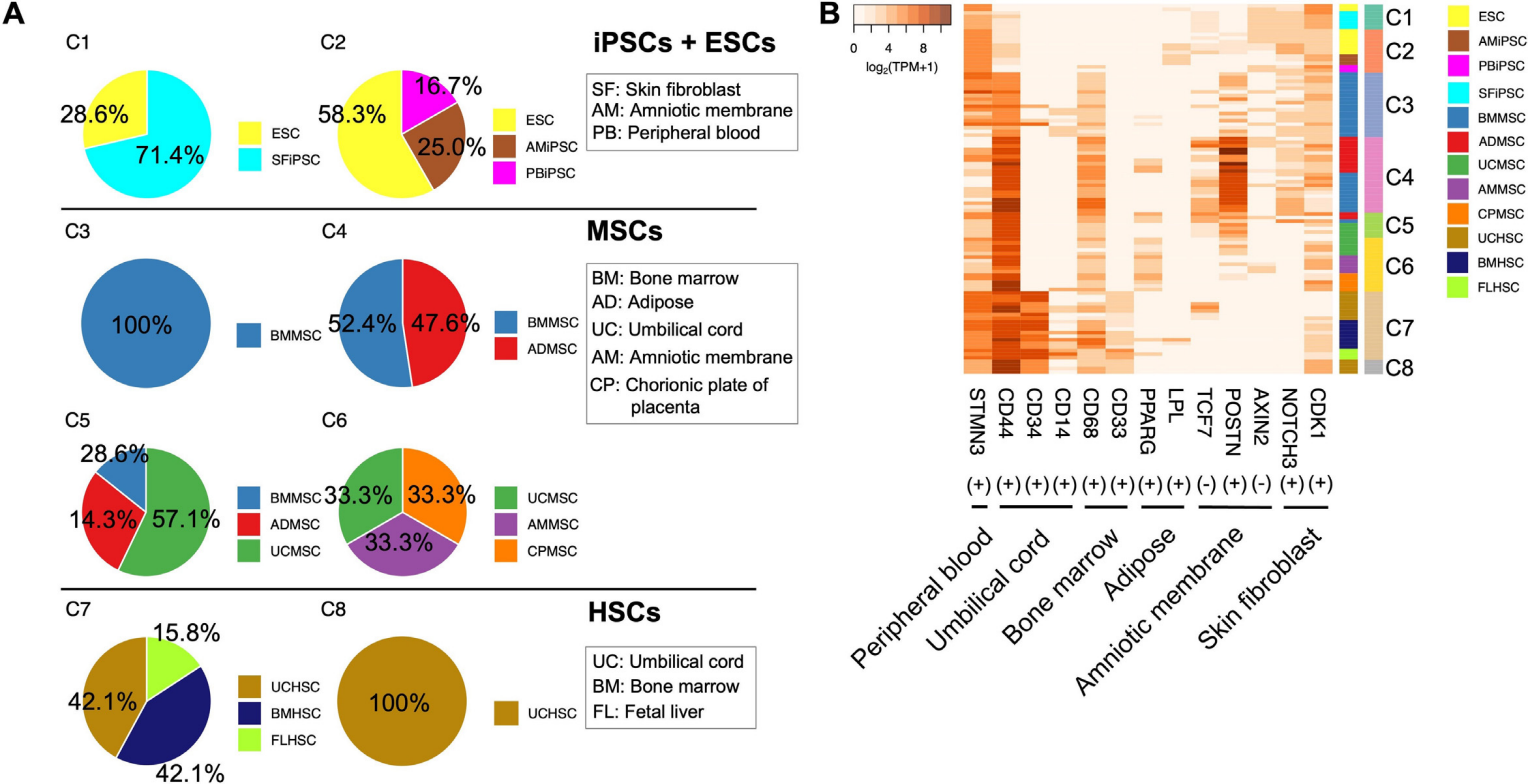
Effects of tissue origin on the cluster classification and the gene expression profiles among stem cells. (**A**) The pie charts show the content of cells for each tissue origin in each cluster. (**B**) Heatmap of the transcription level of positive (+) or negative (-) tissue-specific genes (in log2 of TPM). The darker orange color represents higher expression levels. Along the *x*-axis, tissue-specific genes are shown. The two color bars along the y-axis represent the stem cell tissue origins (left) and the stem cell clusters (right).

We investigated the correspondence between the stem cells in the eight clusters and their tissue origins (Figure 3A). Three types of iPSCs (SFiPSCs, AMiPSCs and PBiPSCs, where SF, AM and PB means skin fibroblast, AM means amniotic membrane and PB means peripheral blood) were classified somewhat corresponding to their tissue origins: C1 contained SFiPSCs (*n* = 5), while C2 contained AMiPSCs (*n* = 3) and PBiPSCs (*n* = 2). Interestingly, ESCs that were commonly derived from the inner cell mass were separately grouped into C1 (*n* = 2) and C2 (*n* = 7). We further analyzed the expression pattern of their tissue lineage-specific genes (Figure 3B). The identifiers were listed in Supplementary Table S4. In SFiPSCs and PBiPSCs, marker genes for skin fibroblasts (CDK1 and NOTCH3) and those for peripheral blood (STMN3) were highly expressed in the cells that originated from the corresponding tissues, respectively. On the other hand, in AMiPSCs, positive expression of a marker gene for amniotic membrane (POSTN) was not observed unlike previously reported (75), although negative expressions of AXIN2 and TCF7 were consistent with previous findings (76,77). Apparently, two subsets of ESCs belonging to C1 and C2, respectively, showed a similar expression pattern to those of iPSCs in each cluster. In HSCs, hematopoietic lineage-specific genes, especially bone marrow-related ones (CD68 and CD33), umbilical cord-related ones (CD14, CD34 and CD44) and peripheral blood-related STMN3 exhibited remarkably high expression. However, in bone marrow-related or umbilical cord-related genes, there was no clear correspondence between the originated tissues (BM and UC) and their expression patterns. In contrast with iPSCs and HSCs, the correlation between the originated tissues and the expression level of their marker genes was rarely observed in MSCs. In particular, an amniotic membrane-related gene POSTN, an adipose-related gene PPARG, a bone marrow-related gene CD68, and an umbilical cord-related gene CD44 showed relatively high expression in clusters that contain unrelated MSCs. In summary, we showed the relative strength of tissue origin impact on their specific gene expression in various types of stem cells: iPSCs tend to be dependent on their tissue origins; ESCs, derived from a single tissue, are split and fused into two clusters of iPSCs; HSCs commonly exhibit high dependency on their tissue origins; MSCs tend to exhibit less dependency on their tissue origins compared with the others.

### The effects of culture conditions in each stem cell type

**Figure 4.**
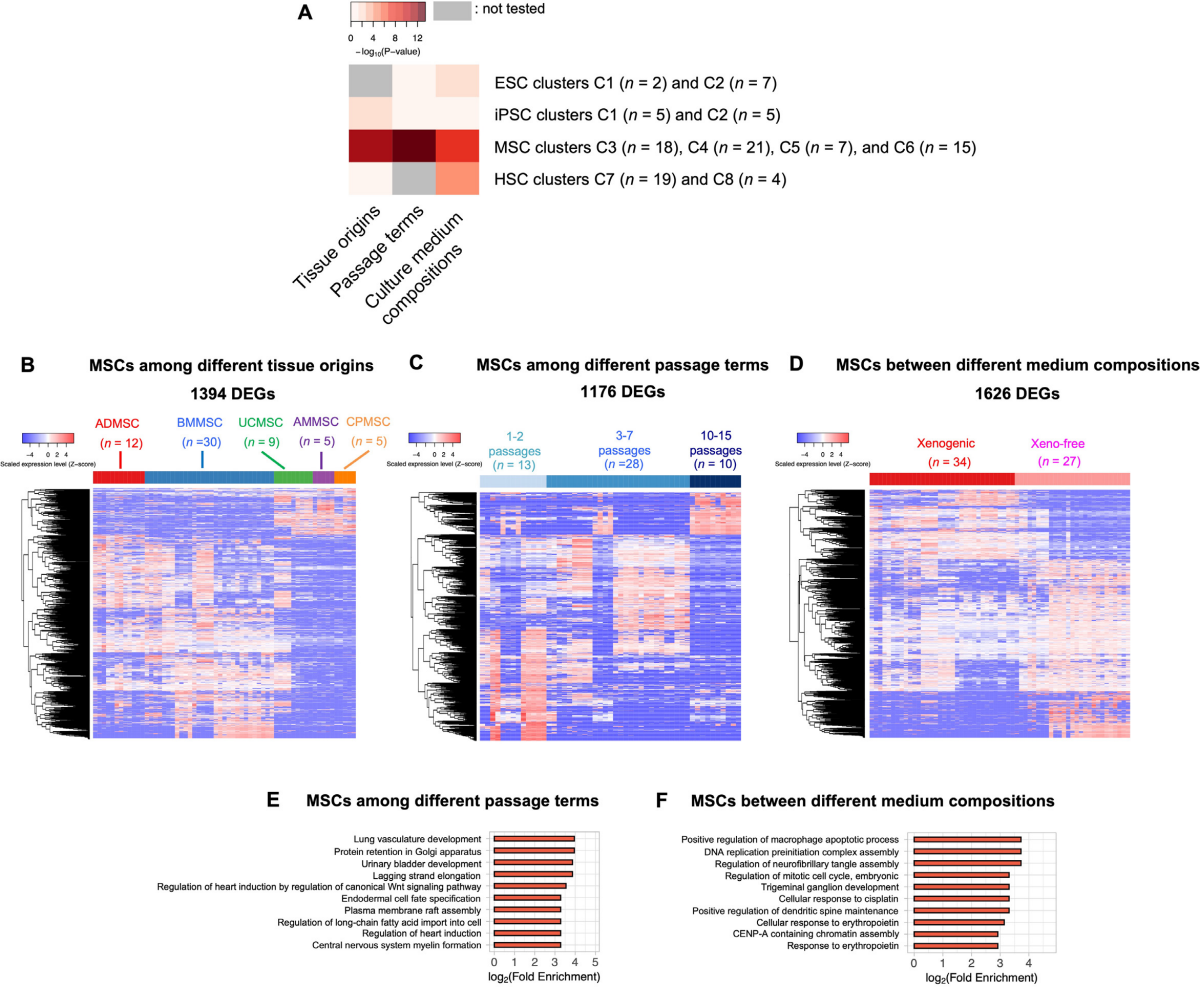
Effect of various factors on the clustering. (**A**) The heatmap shows -log10 of the *P*-value significance of the interrelation between the cluster classification and each factor in each stem cell type. *P*-values were calculated with the chi-square test. The *x*-axis and the *y*-axis represent the factors and the stem cell clusters with the number of stem cell samples, respectively. The darker red color represents lower *P*-values. The gray color represents the interrelation not tested due to a lack of annotations. (**B**) Heatmap of DEG expression profile among MSCs with 12 different stem cell tissue origins. (**C**) Heatmap of DEG expression profile among MSCs cultured in three passage terms (1–2, 3–7 and 10–15 passages). (**D**) Heatmap of DEG expression profile between MSCs cultured in xenogenic and xeno-free medium composition. Heatmaps (B–D) show Scaled expression levels (Z-score). The red color represents higher expression levels, and the blue color represents lower expression levels, respectively. The color bar on the *x*-axis shows the MSC groups. DEGs shown on the *y*-axis are hierarchically clustered. The top 10 enriched GO terms (*P* < 0.01 and fold enrichment ≥2) in the DEGs detected from MSCs with the different passage terms (**E**) and (**F**) with the different medium compositions.

We explored significant (*P* < 0.05) relationships between the stem cell clustering and various annotated factors, using a component of the R package, swamp that was used to test the significance of PCC (Figure 4A). The annotations of the culture composition and the passage of all stem cell replicates are listed (Supplementary Table S1) and visualized (Supplementary Figure S2A and B). In iPSCs, their tissue origin showed the most significant (*P* < 0.01) interrelation while the passage and the culture medium composition were not significant (*P* > 0.05). As shown in Supplementary Figure S2B, iPSCs were classified with different passages as well as their tissue origins. About 50% of iPSCs with 26 and 10 passages were included in C1 and C2, respectively, while the other half was not annotated with their passage information. In ESCs, the culture medium composition (*P* < 0.05) showed the most significant interrelation: their separated clusters corresponded to the xenogenic medium-cultured group (C1) and the xeno-free medium-cultured group (C2). In HSCs, the culture medium composition was significant (*P* < 1 × 10^−4^) while the tissue origin was not highly related to the HSC clustering: the two HSC clusters were characterized as the group cultured in the L-GLN-containing medium (C7) and those cultured in the L-GLN-free medium (C8). In sum, a single factor was highly interrelated with the clustering of iPSCs, ESCs and HSCs, respectively. On the other hand, multiple factors including the tissue origin (*P* < 2 × 10^−11^), the passage term (*P* < 4 × 10^–13^) and the culture medium composition (*P* < 6 × 10^−8^) showed high interrelations with the MSC clustering. The passage term was the most significant interrelation in MSCs: C3 contained the largest ratio of long passage (10–15 passage) group (55.6%), 42.8% of C4 were the middle passage (3–7 passage) group (ADMSCs: 50% and BMMSC: 36.4%), and all replicates in C5 and C6 were the short passage (2 passages) group and the middle passage (3 and 5 passages) group, respectively.

Next, we explored DEGs (differentially expressed genes defined by FDR < 0.05 and |FC| ≥ 2) from each stem cell type to investigate which genes were affected by factors in their expression profiles more strongly. From independent iPSC microarray data, more than six times as many DEGs (467 genes) were detected between iPSCs with different tissue origins than the DEGs (71 genes) detected among iPSCs with different passage terms (Supplementary Figure S3A and B). We investigated DEGs between each of the other stem cell groups (ESCs, MSCs or HSCs) with their significantly correlated factors (Supplementary Table S1). As the result, 698 DEGs were detected between ESCs with different medium compositions (Supplementary Figure S3C), as well as 1775 DEGs between HSCs with them (Supplementary Figure S3D). Different gene functions were enriched in DEGs detected from ESCs and DEGs with different medium compositions, respectively. DEGs from the ESCs were enriched in angiogenesis and cell proliferation related to neurogenesis (Supplementary Figure S3E and Supplementary Table S5A), whereas DEGs from the HSCs were enriched in histone modification (Supplementary Figure S3F and Table S5B). We further explored highly significant correlations of multiple factors by detecting DEGs among MSCs with different tissue origins or culture conditions. As shown in Figure 4B–D, although the number of DEGs from MSCs with different passage terms (1176 genes) was less than that from MSCs with different medium compositions (1626 genes) or tissue origins (1394 genes), the differential expression pattern was more remarkable in MSCs with different passage terms as well as with different medium compositions. DEGs from MSCs with different passage terms were enriched in mesoderm lineage tissue-related functions including kidney development and myogenesis (Figure 4E and Supplementary Table S5C). In contrast, DEGs from MSCs with different medium compositions were not enriched in specific-lineage tissue functions, but they were enriched in cell-cycle and DNA replication (Figure 4F and Supplementary Table S5D). Thus, our data imply that the major factors for the classification of stem cell types are the tissue origin and the culture medium composition (particularly the xeno-free condition) in iPSCs and ESCs, respectively (note that the information on culture conditions was obtained only insufficiently in iPSCs); the culture medium composition (particularly the L-GLN condition) in HSCs; and the passage term in MSCs, though the influence of some other factors cannot be ignored.

### Identifying characteristic genes for the stem cell clusters

**Figure 5.**
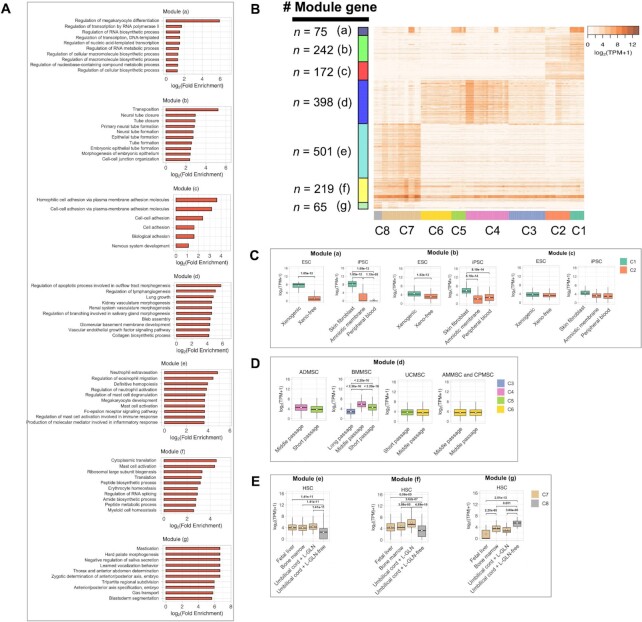
Identifying characteristic genes for the stem cell clusters. (**A**) The top 10 enriched GO terms (*P* < 0.01 and fold enrichment ≥2) in the seven modules. (**B**) Expression profile of the genes that characterize the stem cell clusters. The heatmap shows the expression of co-expressed genes (in log2 of TPM). The darker orange color represents higher expression levels. Color bars along the *x*-axis and the *y*-axis represent the modules with the number of their genes and the stem cell clusters, respectively. (**C–E**) Differential gene expressions of each module among and within clusters in pluripotent stem cells, MSCs, and HSCs, respectively. The expression level of DEGs (|FC| ≥ 2 and *P* < 0.01) is shown in log2 of TPM. The open circle in each box represents the mean expression level. The *P*-value (above each box) was calculated from Tukey’s multiple comparison test with ANOVA.

Next, we identified genes characteristic of each stem cell cluster by WGCNA. Seven modules designated as (a)–(g) were significantly co-expressed with stem cell clusters (Table 2 and Supplementary Table S7). We functionally characterized the modules through the gene functional enrichment analysis. The top 10 enriched gene ontology (GO) terms (*P* < 0.01 and fold enrichment ≥2) were extracted from each module (Figure 5A and Supplementary Table S8). Then, we showed the expression profile of the seven modules (Figure 5B), in which we observed stem cell type-specific expression patterns of Modules (b)–(g). Most of these modules were enriched in reasonable tissue lineage-related functions, such as morphogenesis of neural and embryonic epithelium lineages in pluripotent stem cell-specific Module (b), mesoderm lineage development in MSC-specific Module (d) and immune response, hematogenic functions, and the processes during early development in HSC-specific Modules (e)–(g). Moreover, Module (a) showed remarkably high expression in pluripotent stem cell cluster C1 (Figure 5B) with a significant correlation between the module and the cluster (Table 2). However, subsets of stem cells contained in MSC clusters (C5 and C4) and HSC cluster C7 also showed high expressions of Module (a) as well as C1. This module was largely enriched in terms of ubiquitous functions, including transcriptional regulation and biosynthesis. Then, we explored the expression levels of stem cell type-specific modules that correspond with the DEGs among clusters to extract genes that exhibit characteristic expression. In pluripotent stem cell clusters, both Modules (a) and (b) were highly expressed in C1 (the xenogenic medium-cultured group of ESCs and SFiPSCs) compared to C2 (the xeno-free medium-cultured group of ESCs, AMiPSCs, and PBiPSCs), whereas Module (c) was not differentially expressed between the two clusters (Figure 5C). In MSC clusters, Module (d) exhibited higher expression in C4 (middle-passage term group) than in other clusters, and its expression was particularly clear in BMMSCs (Figure 5D). In HSC clusters, both Modules (e) and (f) were highly expressed in C7 (cultured in the medium containing L-GLN) compared to C8 (cultured in an L-GLN-free medium), whereas the opposite expression change was observed for Module (g) (Figure 5E).

**Table 2.** Gene co-expression modules which are characteristic of the stem cell clusters with their attributes. ‘*r*’ represents Pearson's correlation coefficient

Module	#Gene	Highly correlated stem cell cluster	*r*	*P*-value
(a)	75	Pluripotent stem cell cluster (C1)	0.79	8.00E-23
(b)	242	Pluripotent stem cell cluster (C1)	0.93	5.00E-47
(c)	172	Pluripotent stem cell cluster (C2)	0.74	3.00E-19
(d)	398	MSC cluster (C4)	0.72	8.00E-18
(e)	501	HSC cluster (C7)	0.93	5.00E-47
(f)	219	HSC cluster (C7)	0.71	3.00E-17
(g)	65	HSC cluster (C8)	0.92	7.00E-43

### Differential expression of histone genes across multiple stem cell types

**Figure 6.**
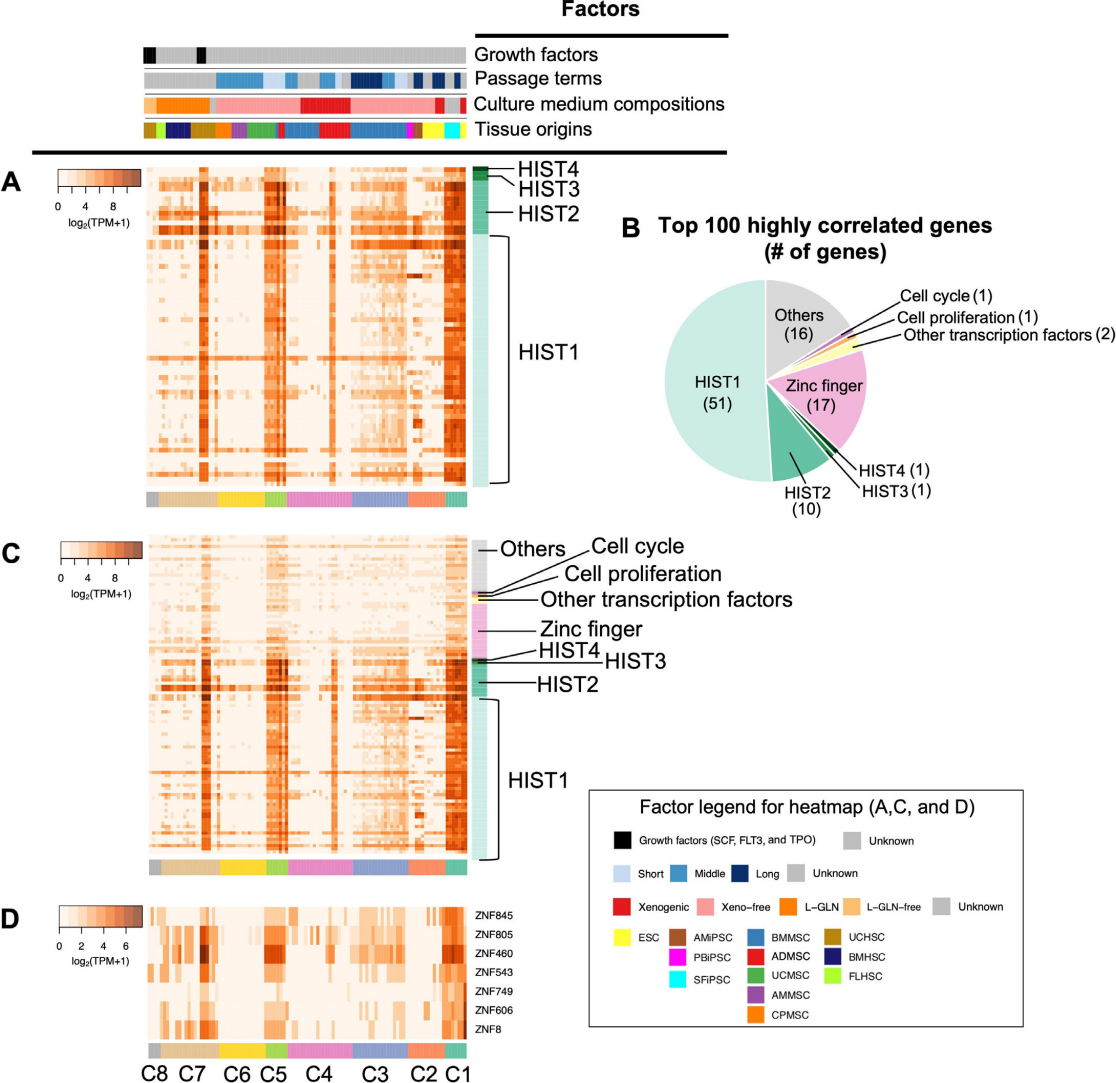
Differential expression of histone genes depending on the culture conditions across multiple stem cell types. (**A**) Heatmap showing the expression of 66 histone genes (in log2 of TPM). The darker red represents higher expression levels. The color bar along the *y*-axis represents the distinction of 66 histone genes based on their gene families (HIST1–4). The color bars along the *x*-axis represent the four kinds of annotated factors (top) and the stem cell clusters (bottom). (**B**) The functional classification of the top 100 genes highly correlated with the expression pattern of the histone gene set. (**C**) Heatmap showing the expression of the top 100 genes highly correlated with histones. (**D**) Heatmap of expression profile for chr19 co-localized seven genes coding ZNFs, which are included in the top 100 genes. Heatmaps (A, C and D) show the expression level (in log2 of TPM). The darker orange color represents a higher expression level. Genes are arranged along the *y*-axis. Color bars along the *x*-axis represent four kinds of annotated factors (top) and the stem cell cluster (bottom of each heatmap), respectively.

Lastly, we focused on Module (a) in which quite a high expression was observed among three modules: (a), (d) and (f), which exhibited high expression patterns across multiple stem cell types (Figure 5B and Supplementary Figure S5). To our surprise, we found that 51 genes in this module were histone genes (corresponded with 78.5% of genes in the module). These genes correspond with 70.8% of known canonical histone genes (i.e. replication-dependent histone genes) (87,88). Thus, the specific expression pattern of Module (a) is mainly caused by the high expression level of histone genes. In the human genome, canonical 72 histone genes are located within four histone clusters (HIST1–4) (87), where HIST1 on human chromosome 6 is the largest family. All 66 of the canonical histone genes belonged to HIST1–4 (78.8% were in HIST1) (Figure 6A). We checked if there are any factors that are well correlated with the specific expression of these 66 histone genes. C1 (ESCs of the xenogenic medium and SFiPSCs) and C5 (MSCs of both the short-passage and xeno-free medium) showed commonly high expression. Interestingly, additional stem cell samples in MSCs and HSCs, which were cultured in specific culture conditions (Supplementary Table S1), showed higher expression of these histone genes. MSCs of a high-glucose medium in C4 exhibited higher expression than that of a low-glucose medium, as well as HSCs of the medium with L-GLN and three growth factors (SCF, FLT3 and TPO) in C7 than that of the medium with either L-GLN or the growth factors. Additionally, we investigated histone expression patterns among mouse undifferentiated stem cells, by analyzing publicly available mouse RNA-seq data (Supplementary Table S9). We conducted the analysis through the same process as done in the human RNA-seq dataset, including batch effect correction (Supplementary Figure S6A and B), dimensionality reduction, stem cell clustering (Supplementary Figure S6C and D), and expression analysis for 41 kinds of mouse histone genes corresponded with human orthologs (Supplementary Figure S6E). We observed similar features of those mouse histone expression patterns with that shown in human histones: high expression in ESCs with the xenogenic medium, iPSCs derived from fibroblasts, and HSCs of medium with L-GLN or glucose (Supplementary Figure S6E).

Previous studies reported that histone genes and cell proliferation-related genes were co-expressed during the cell cycle (89,90). However, in our result, gene sets related to the regulation of cell cycles (GO:0051726, 1087 genes) and the population proliferation (GO:0042127, 1799 genes) neither showed similar expression patterns with the histone gene set (Supplementary Figure S7A and B). Thus, we attempted to explore whether human histone genes are highly correlated with other genes. We investigated the correlation of expression patterns between the 66-histone gene set and all individual genes. We extracted the top 100 genes whose expression patterns showed the highest correlation with that of 66 histone genes (Figure 6B and C and Supplementary Table S10). 17 zinc finger family genes, including 16 ZNF genes, were the second highest ratio in the extracted genes (next to 51 HIST1 cluster genes). Notably, 11 ZNF genes of them were commonly located in chromosome 19, in which seven genes (Figure 6D), including ZNF845, ZNF805, ZNF460, ZNF543, ZNF749, ZNF606 and ZNF8, were within 19q13.42 - 19q13.43. Lastly, we confirmed that stem cell clustering using a dataset without histones and top 100 genes highly correlated with histones (Supplementary Figure S8) showed a similar classification to that shown in Figure 2, suggesting these genes were not strongly influenced by overall gene expression profile. Altogether, we showed that the histone gene set and their highly correlated genes including the co-localized zinc finger family exhibited high expression patterns in undifferentiated human stem cells with specific culture conditions, which was similarly observed in undifferentiated mouse stem cells, especially, in mouse ESCs.

## DISCUSSION

Through our comprehensive comparison of multiple types of undifferentiated human stem cells, we ranked the relative importance of multiple factors, such as their tissue origins and the culture conditions, to stem cell gene expression profile. Our result of clustering by UMAP showed a larger diversity of the expression profile consisting of 9679 genes among somatic stem cells than that among pluripotent stem cells (Figure 2). HSCs and MSCs exhibited distinguishable expression profiles, whereas ESCs and iPSCs were not separated clearly in terms of their expression profiles. These results suggest that expression profiles at the transcriptome level are largely affected by stem cell types and their pluripotency.

Based on the stem cell clustering result (Figure 2), we further explored which genes were affected by factors in expression profiles among undifferentiated stem cell types more strongly (Figures 3–5). We observed that ESCs and iPSCs were divided into two clusters, where iPSCs were highly correlated with their tissue origins while ESCs with the xenogenic/xeno-free conditions in their culture medium (Figure 4A). In our analysis, the impact of tissue origins on the tissue origin-related gene expressions was stronger in iPSCs than in other stem cell types (Figure 3). Our comparison using independent microarray data of iPSCs showed that more diversity was observed between different tissue origins (Supplementary Figure S3A) than among different passage terms (Supplementary Figure S3B), implying that tissue origins have a relatively large effect on the gene expression profile of iPSCs. In ESCs, we found that DEGs between different medium compositions were enriched in neuronal cell proliferation (Figure S3E). This functional enrichment related to neurogenesis was similarly observed in WGCNA. In the pluripotent stem cell cluster which includes ESCs cultured in xenogenic culture media and SFiPSCs, genes related to embryonic epithelial morphogenesis and neural tube development were highly expressed (Figure 5A–C). These results were supported by previous findings reporting that dermal fibroblast-derived iPSCs and ESCs show similar neuronal potential (91) and the differentiation potential of stem cells can be significantly affected by the difference in culture medium ingredients (92,93).

Another culture medium composition on the L-GLN condition was significantly correlated with HSC clusters. We found that DEGs detected from HSCs with the L-GLN /L-GLN-free conditions in their culture medium were enriched in histone modification (Supplementary Figure S3F). Moreover, as shown in Figure 5A, B and E, three modules were strongly involved in HSC clusters. HSCs with L-GLN medium composition highly exhibited somewhat enriched in immune and hematopoietic features reflecting their tissue origins, which is consistent with previous results (94,95), while HSCs with L-GLN-free medium composition highly exhibited genes related to early development, implying that undifferentiated HSCs possess pluripotent cell-like identity or specific tissue lineage such as immune and hematopoietic cells.

In contrast with two types of pluripotent stem cells and HSCs, the clustering of MSCs was rather weakly influenced by their tissue origins on the tissue origin-related gene expressions (Figure 3). This suggested a relatively weaker impact of tissue origins on MSCs than that on other stem cell types. However, within MSC clusters, multiple factors including both tissue origin and culture conditions, including passage terms and medium compositions, were significantly correlated (Figure 4A). In addition, the differential expression patterns of DEGs from MSCs with different culture conditions were more remarkable (Figure 4C and D) than that of DEGs among five kinds of different tissue-derived MSCs (Figure 4B), although tissue origins were involved in the more number of DEGs than passage terms. These observations suggested that tissue origin is one of the major factors that provide many DEGs among undifferentiated MSCs, however, its impact in MSCs is not as strong as impacts in other stem cell types or impacts of culture conditions in MSCs. In further analysis, we found that DEGs from MSCs with different passage terms were enriched in mesoderm lineage tissue-related functions including kidney development and myogenesis (Figure 4E), whereas enriched functions related to lineage tissues were not detected from that with different medium compositions (Figure 4F). Similarly, the module specifically correlated with the MSC cluster was enriched with genes related to multiple mesoderm-lineage developments (Figure 5A, B and D). MSCs derived from bone marrow with middle passages exhibited significantly distinct expressions. Considering that MSCs cultured in 5 or 10–14 passages exhibit fewer senescence levels and increased proliferation activity (96,97) despite they generally enhance the stem cell senescence with increased passages (98–100), we suggest that MSCs cultured in middle passages are in an activated state with the increasing identity related to multiple mesoderm lineages. Altogether, factors that characterize stem cell types strongly affect their differential expression of tissue lineage genes, and subsequently might alter their undifferentiated stem cell identity.

In addition to the stem cell type-specific modules that are strongly related to the factors governing the distinction of cell types, such as tissue origins and culture conditions, we identified a number of histone genes (located in the HIST1–4 clusters) that exhibit higher expression levels across various cell types which are characteristic with their culture conditions, such as the short-passage term, the addition of growth factors and the use of nutrient-rich media (Figure 6A). These conditions are supposed to enhance cell growth and activity, as previously reported (100–102). We confirmed a similar tendency with mouse histone genes (Supplementary Figure S6E), especially in particular stem cells (fibroblast-derived iPSCs) or culture conditions (xenogenic media and medium with L-GLN or glucose), suggesting some conserved feature in histones between human and mouse undifferentiated stem cells although the expression data and their factor annotations in mice were not plenty as those in humans. We further detected 16 ZNF genes that exhibit a co-expression pattern with the histone genes in the specific stem cell and growth conditions (Figure 6B and C), of which seven genes were closely located within chromosome 19 (13q42-13q43) (Figure 6D). Previous studies reported the connection between stem cell identity and histones or zinc fingers: the activation of multiple histone variants, including H1, H2A and H3, in maintaining ESC pluripotency and ESC fate (103,104), the role of histone variant H3.3 on the maintenance of HSC stemness (105), and co-localized 31 zinc-finger genes within topologically associating domains on chr19 in human ESCs (106). We found a higher co-expression pattern between histones and co-localized zinc fingers in ESCs in xenogenic medium and HSCs in L-GLN medium (Figure 6A and C). In particular, the difference in medium compositions in HSCs was involved in differential expression of genes related to histone modification (Supplementary Figure S3F), implying the importance of culture conditions in controlling stem cell identity via co-expression between histones and zinc fingers. Further experimental analyses on the association of culture conditions with controlling stem cell identity would be required.

**Figure 7.**
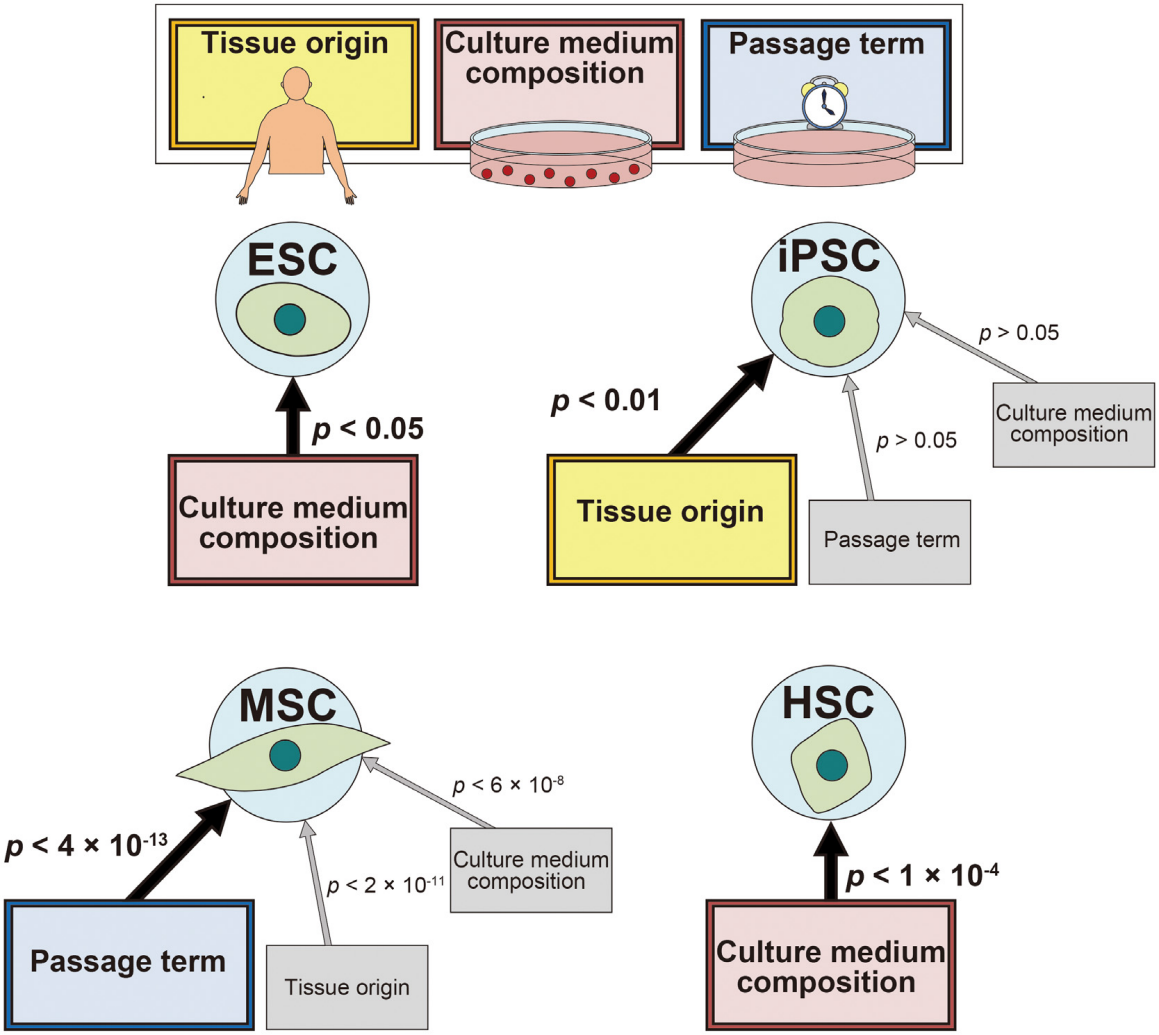
The relative impact of biological and technical factors among undifferentiated human stem cell types. We ranked the important factors (tissue origin, culture medium composition and passage term) influencing gene expression in four types of undifferentiated human stem cells (ESCs, iPSCs, MSCs and HSCs). The illustration shows the major factor(s) in each stem cell type and the relative strength of its impacts with the *P*-value significance of the interrelation between the stem cell type clustering and each factor. The factor with a larger rectangle and arrow represents a relatively stronger impact on the stem cell type while one with a smaller rectangle and gray arrow has a relatively weaker impact.

Altogether, we ranked the impact of biological and technical factors among undifferentiated human stem cell types. We characterized the major factor in each stem cell type and identified the relative strength of its impacts (Figure 7), such as tissue origins in iPSCs and culture conditions in ESCs and HSCs. MSCs exhibited lower strength of their tissue origins than other stem cell types, whereas they were more significantly correlated with culture conditions within multiple factors. Moreover, the impact of culture medium composition on histones and zinc fingers might be highly involved in stem cell identity. These results suggest a new insight into stem cell features, which could allow experimental researchers to have further insight into basic stem cell identity. Our findings would be helpful to evaluate important factors in treating each type of undifferentiated stem cells in regenerative medicine.

## DATA AVAILABILITY

All the data used in this study were downloaded from public data sources. Raw data and scripts for batch effect correction using Risso et al.’s RUVSeq (57) were uploaded to a GitHub repository: https://github.com/yyamatani/2022_scripts_and_data and Zenodo (http://doi.org/10.5281/zenodo.7278583).

## Supplementary Material

lqac087_Supplemental_Files
