# Supplementary material for: Comprehensive comparison of gene expression diversity among a variety of human stem cells
Source: NAR Genom Bioinform. 2022 Nov 29;4(4):lqac087. doi: 10.1093/nargab/lqac087 (PMC9706419; doi:10.1093/nargab/lqac087)
Supplement: lqac087_Supplemental_Files [file lqac087_supplemental_files.zip › Revised_Supplementary_Data2.docx]

**Supplementary Data**

**Supplementary Method S1.** Parameter choice of UMAP.

Three parameters of UMAP were selected: (i) dimensions to use as input features (*dims* = 5), (ii) the number of neighboring points (*n.neighbor* = 10) used in local approximations of manifold structure and (iii) the resolution parameter (*resolution* = 1.1) for clustering. In setting parameter (i), we determined dimensions within 30 principal components (PCs) that we selected in the preliminary PCA step. We selected 30 PCs based on a rule of thumb and detected significant PCs to use for dimensions. ‘Elbow plot’ (Supplementary Figure S2A) showed a ranking of principal components based on the percentage of standard deviation explained by each one. We can choose the first 5 PCs that kept higher standard deviations, suggesting that the majority of true signal is captured in these PCs. Thus, we have been justified in choosing the number of PCs (PC1-5) to use for dimensionality reduction and set as dimension parameter (*dims* = 5). In stem cell clustering using 103 bulk RNA-seq data samples, we aimed to obtain a greater number of clusters because the number of data samples would be relatively larger in bulk RNA-seq analysis. For this reason, we set parameter (ii) to 10, which is smaller than its default parameter of 15, and parameter (iii) to 1.1, which is larger than its default parameter of 0.8.

**Supplementary Method S2.** Parameter choice of WGCNA.

Six parameters of WGCNA were selected: (i) Soft thresholding power 14, for fulfilling the scale-free topology property of the co-expression network, hierarchical clustering parameters (ii) the minimal module size (minClusterSize = 100), (iii) sensitive module detection parameter (deepSplit = 3) and (iv) the cut height (cutHeight = 0.15) to merge similar modules and parameters for filtering genes in modules (v) gene significance (GS) > 0.5 and (vi) module membership (MM) > 0.8. In setting parameter (i), soft thresholding power closer to R^2^ = 0.9, which is the global best fit, should be used for co-expression network construction (Supplementary Figure S4A). The distribution approximately follows a straight line (scale-free R^2^ = 0.88, slope = -1.69), which is referred to as approximately scale-free topology (Supplementary Figure S4B). We set parameter (ii) to 100 and parameter (iii) to 3, which are relatively high, to identify large modules. Then, we chose parameter (iv) to 0.15, corresponding to the correlation of 0.85, to merge modules in gene expression networks that are too close as measured by the correlation of their eigengenes (Supplementary Figure S4C). 17 assigned modules were obtained while 4894 genes with non-specific expression were grouped into one unassigned module, accounting for 50.6% of all genes (Supplementary Figure S4D and Supplementary Table S6). Through detecting higher correlation (|*r*: Pearson’s correlation coefficient| >= 0.7) between the module eigengenes and the stem cell clusters, seven modules designated as (a)-(g), were identified (Supplementary Figure S4E and Supplementary Table S7). We further extracted the most significantly co-expressed genes from the seven candidate modules by setting the filtering. The significant correlation (correlation >= 0.7) between them supported the reliability of filtering parameters (v) to > 0.5 and (vi) to > 0.8 (Supplementary Figure S4F). Finally, 1672 genes included in the seven modules were obtained in total (Table 2).

**
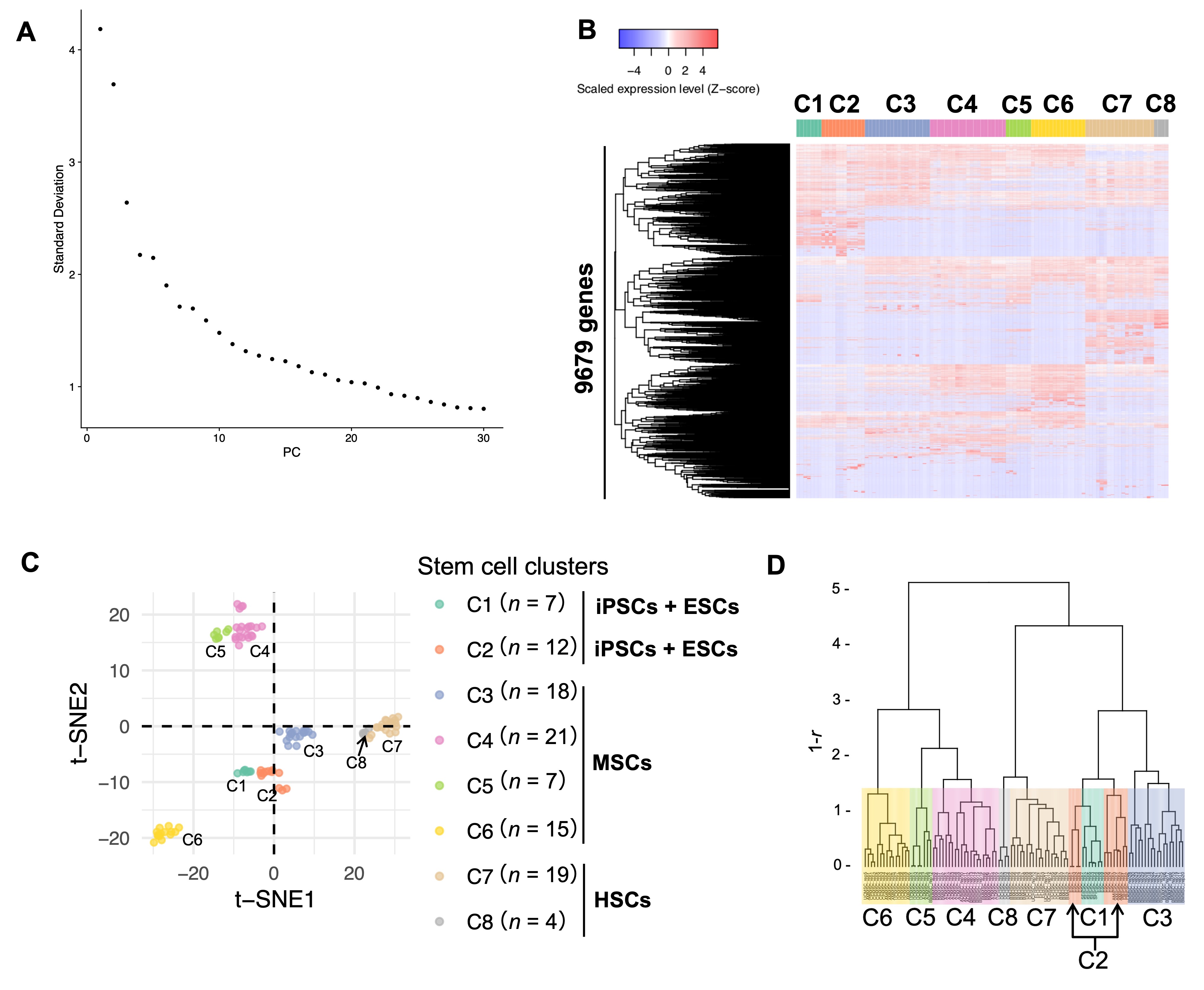
**

**Supplementary Figure S1.** Parameter choice of UMAP and confirmation of the stem cell clustering results. (A) Elbow plot showing a ranking of principal components based on the percentage of standard deviation. (B) Heatmap of gene expression profiles among stem cells. Scaled expression levels (Z-score) of 9679 genes were investigated. The red color represents higher expression levels, and the blue color represents lower expression levels, respectively. The color bar on the x-axis shows the stem cell clusters. Genes shown on the y-axis are hierarchically clustered. (C) Stem cell clustering. The stem cell cluster distribution was obtained using a K-nearest neighbor (KNN) graph, following dimensionality reduction by t-SNE. The individual dot represents each stem cell sample. The colors of the dots are used to distinguish the stem cell clusters (C1-C8). The black arrow points C8. (D) Hierarchical clustering of stem cell samples using Ward’s method. The color panels on the x-axis correspond with those used for the stem cell clusters (C1-C8). “*r*” represents Pearson’s correlation coefficient.

**
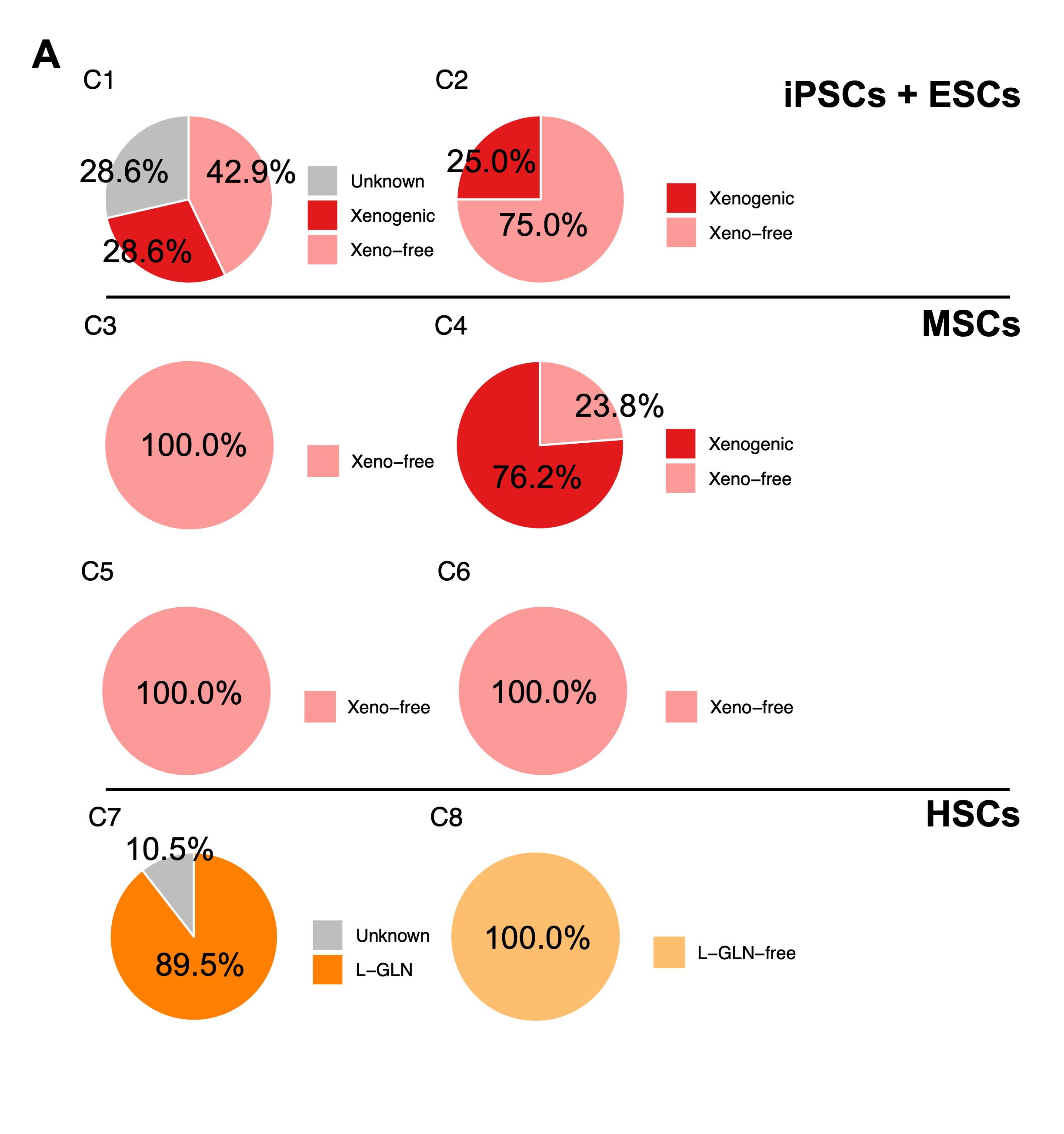
**

**Supplementary Figure S2**

**
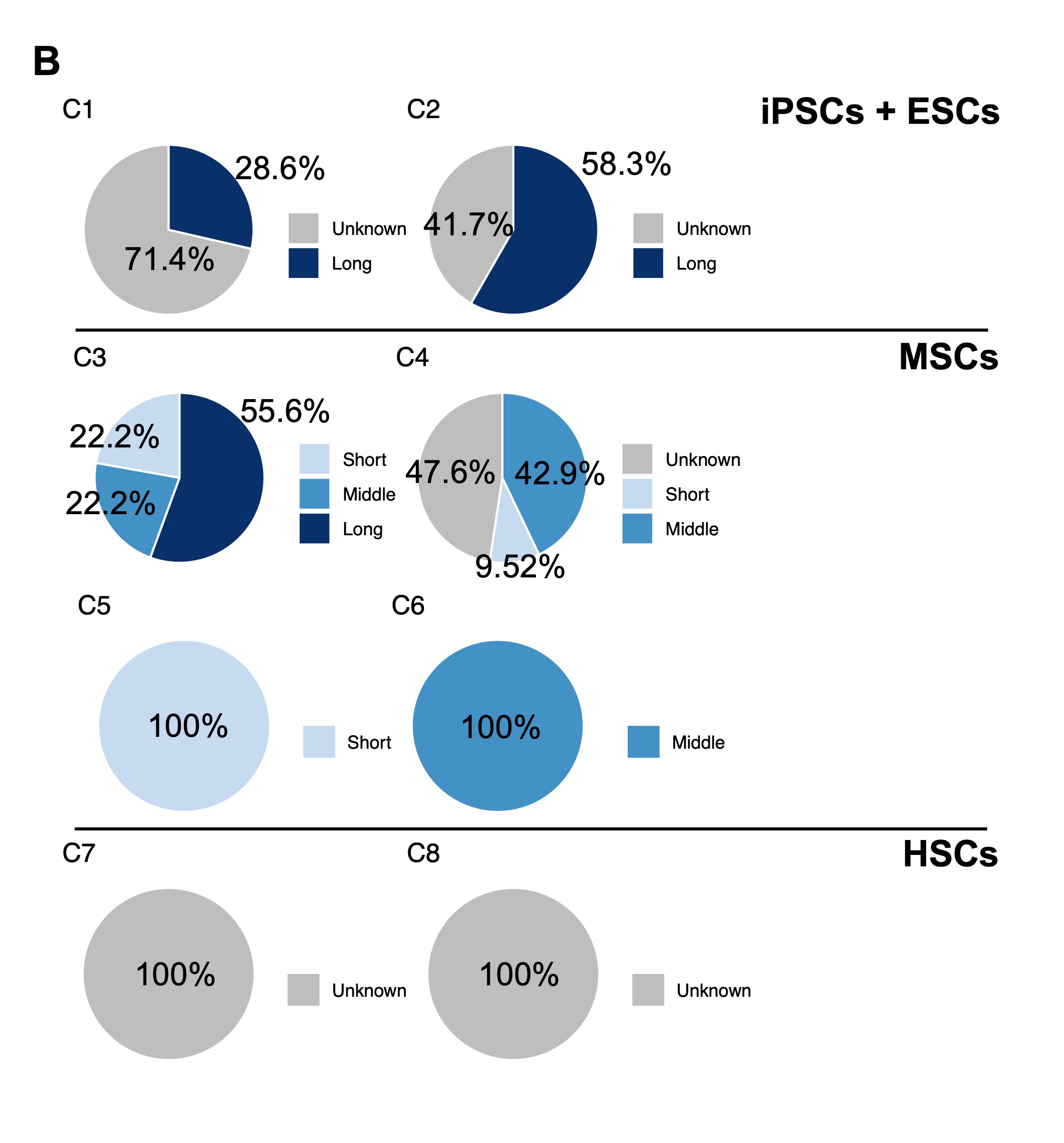
**

**Supplementary Figure S2**

**Supplementary Figure S2.** Characterization of each cluster in terms of factors. The pie charts show the ratio of cells with characteristic (A) culture compositions and (B) passage terms in each cluster.

**
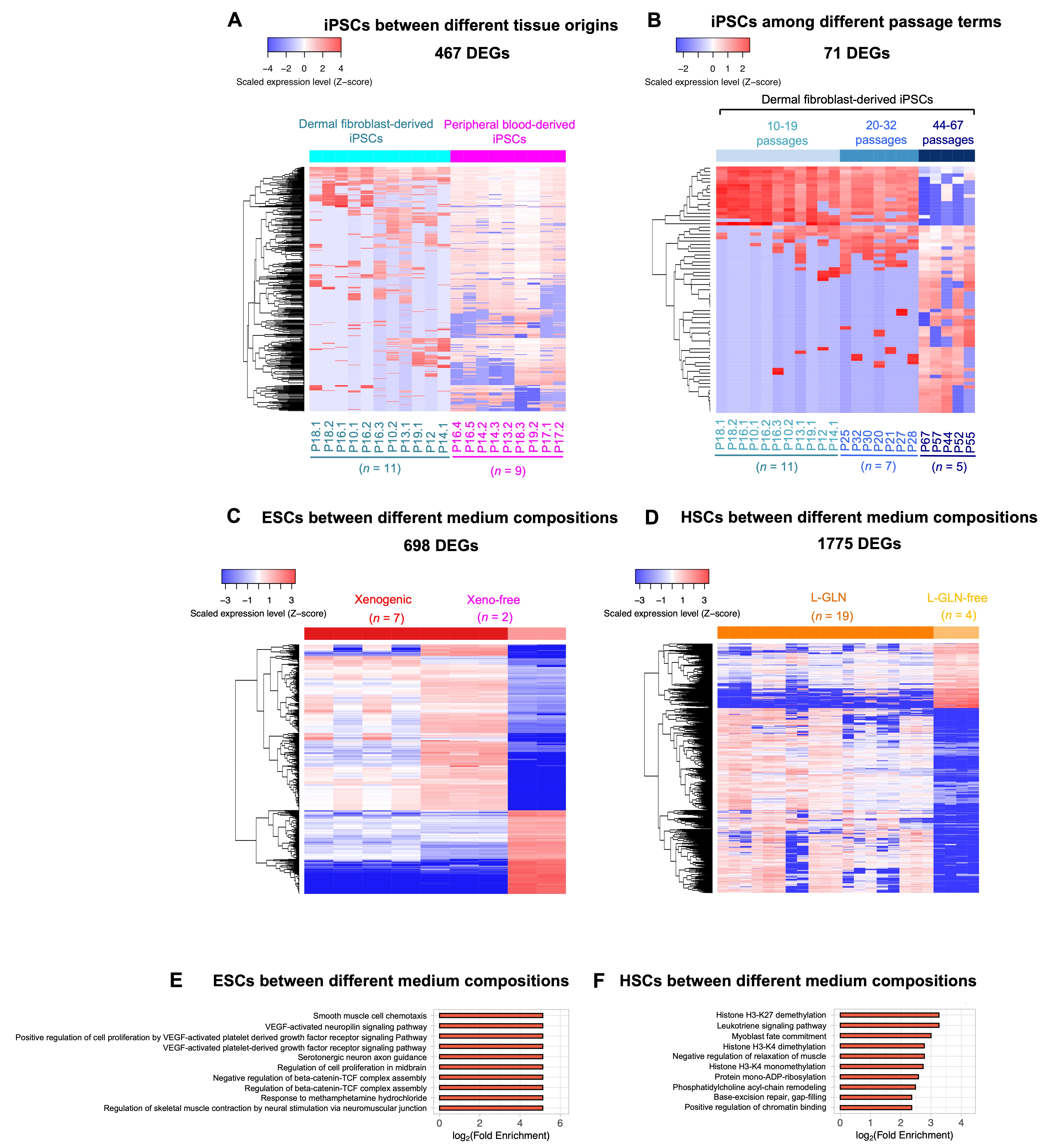
**

**Supplementary Figure S3.** Detection of differentially expressed genes (DEGs) from iPSCs, ESCs, and HSCs. (A) Heatmap of DEG expression profile between dermal fibroblast- and peripheral blood-derived iPSCs. (B) Heatmap of DEG expression profile among dermal fibroblast-derived iPSCs cultured in three passage categories (10-19, 20-32, and 44-67 passages). (C) Heatmap of DEG expression profile between ESCs cultured in xenogenic and xeno-free medium composition. (D) Heatmap of DEG expression profile between HSCs cultured in xenogenic and xeno-free medium composition. Heatmaps (A-D) show scaled expression levels (Z-score). The red color represents higher expression levels, and the blue color represents lower expression levels, respectively. The color bar on the x-axis shows the stem cell groups. DEGs shown on the y-axis are hierarchically clustered. The top 10 enriched GO terms (p < 0.01 and fold enrichment >= 2) in the DEGs detected from (E) ESCs and (F) HSCs with different medium compositions.

**
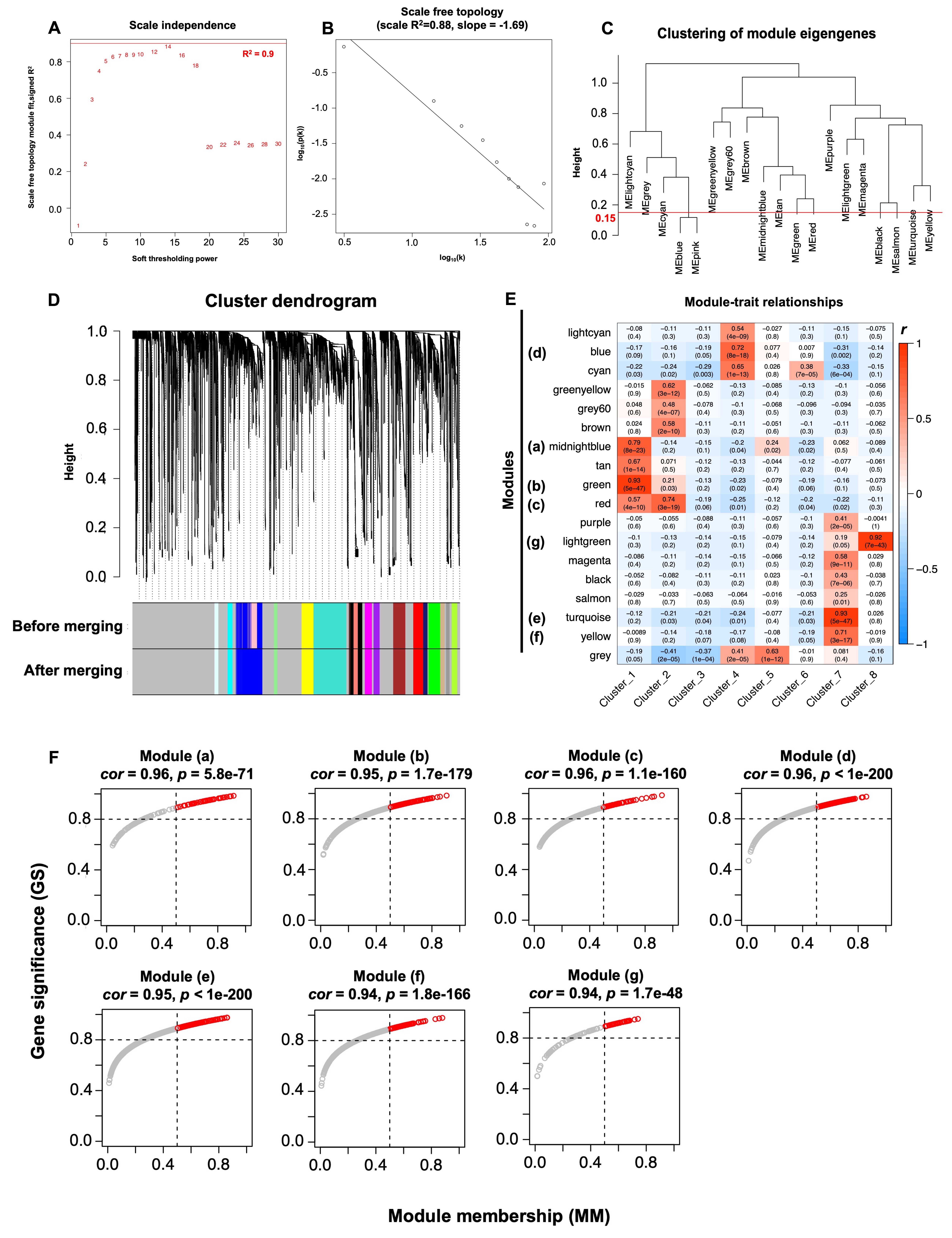
**

**Supplementary Figure S4**

**Supplementary Figure S4.** Confirmation of parameter choice of WGCNA and identification of modules characteristic for the stem cell clusters. (A) the scale-free topology index R^2^ (y-axis) as a function of different soft thresholding power (x-axis). The horizontal line with the red color represents R^2^ = 0.9. (B) Scale-free topology when soft threshold = 14. Linear model fitting of R^2^ index shows the relationship between connectivity and frequency as (k) and p(k), respectively in log10-scaled. (C) Hierarchical clustering dendrogram of the module eigengenes (ME) based on the dissimilarity matrix (1-TOM). Module names correspond with Supplementary Table S6. The horizontal line with the red color represented cut height = 0.15. (D) Clustering dendrogram of genes based on the dissimilarity topological overlap calculation formula (1-TOM). Color labels shown below the dendrogram represent 19 modules before merging and 18 modules after merging. (E) Heatmap of the correlation between the module eigengenes and the stem cell clusters. In each square of the heatmap, the Pearson’s correlation coefficient (top) and P-value (bottom) are shown. Modules with |*r*| >= 0.7 and *p* < 0.05 are considered statistically significant. Seven modules with higher correlations between their module eigengenes and the stem cell clusters were designated as alphabetical identifiers (a)-(g), which corresponded to Supplementary Table S7. (F) Scatter plots of correlation between MM (x-axis) and GS (y-axis) in the seven modules. The correlation and its P-value represent at the top of each plot. The red dots represent genes with GS > 0.5 and MM > 0.8. Dashed lines in each plot represent GS = 0.5 and MM = 0.8, respectively.

**
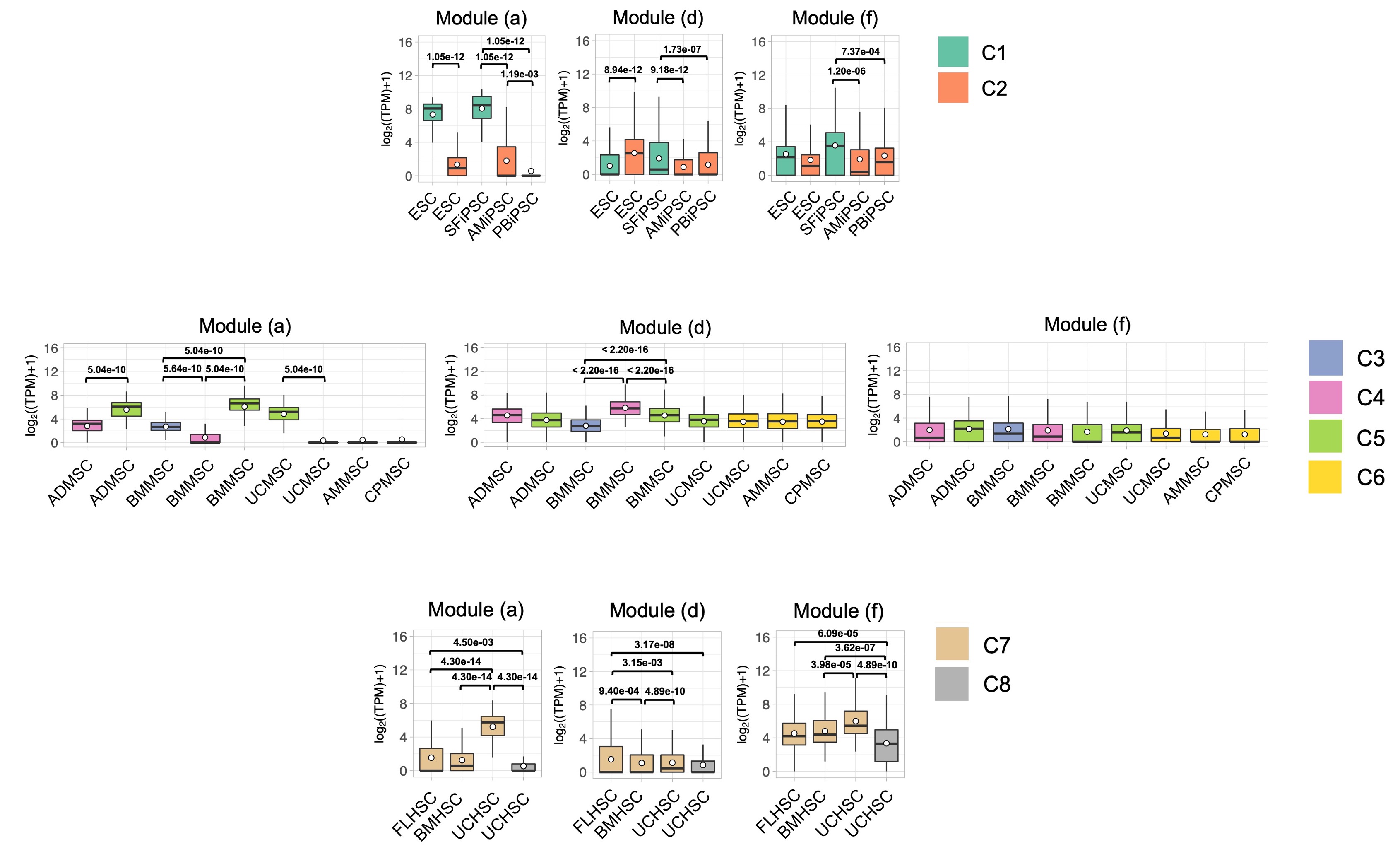
**

**Supplementary Figure S5.** Differential gene expressions of Module (a), (d), and (f) of among and within clusters in pluripotent stem cells, MSCs, and HSCs, respectively. The expression level of DEGs (FC| >= 2 and p < 0.01) is shown in log2 of TPM. The open circle in each box represents the mean expression level. The P-value (above each box) was calculated from Tukey’s multiple comparison test with ANOVA.

**
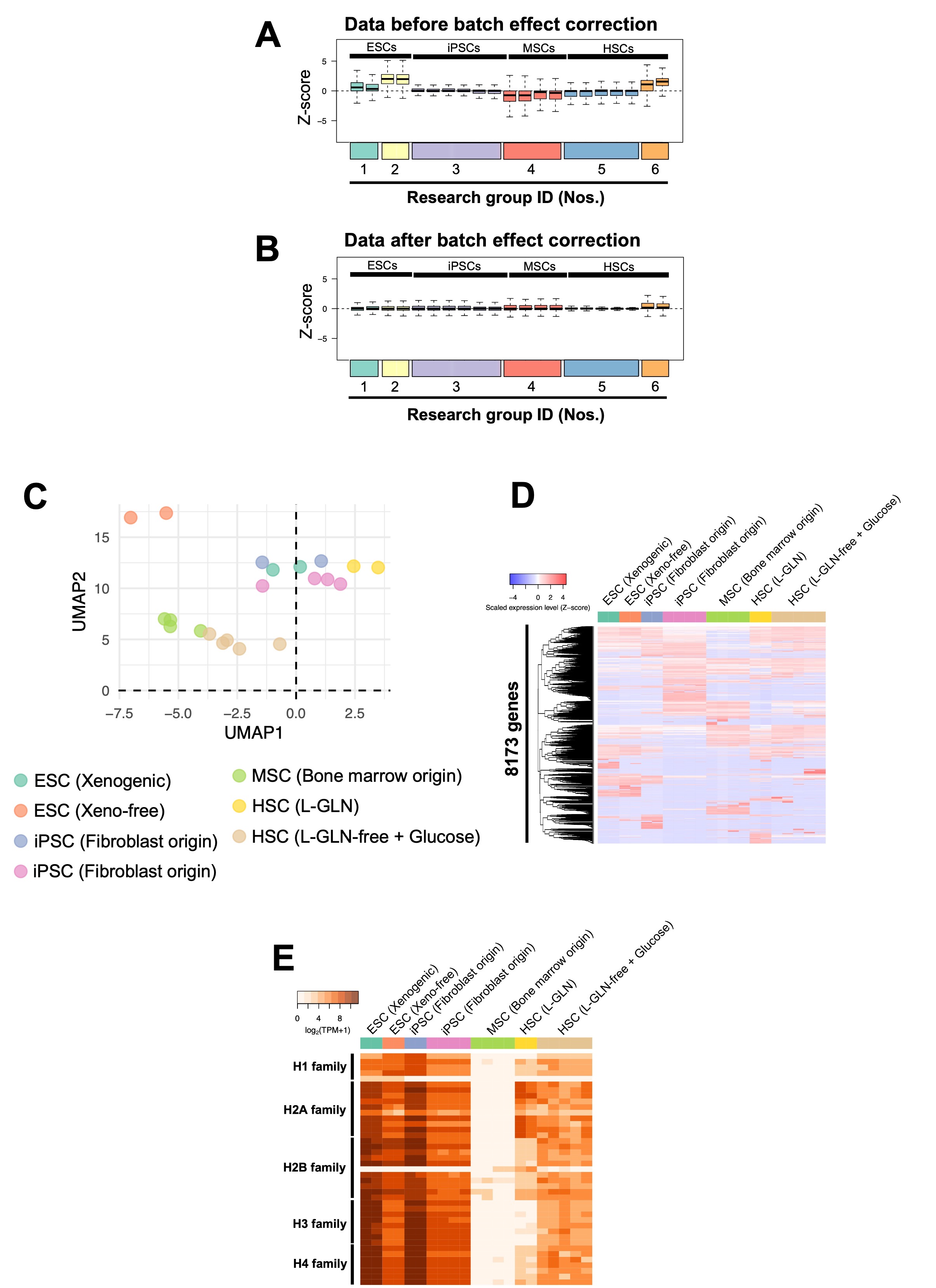
**

**Supplementary Figure S6**

**Supplementary Figure S6.** Mouse undifferentiated stem cell RNA-seq analysis. (A, B) Relative gene expression levels of housekeeping genes (in Z-score) among RNA-seq data (A) before and (B) after the batch effect correction, respectively. The numbers in the horizontal axis show the research groups listed in Supplementary Table S9, and the different colors were used for their easy distinction. (C) The stem cell cluster distribution was obtained using a K-nearest neighbor (KNN) graph with the relative similarity of gene expression profiles among stem cells, following dimensionality reduction by UMAP. The individual dot represents each stem cell sample. The colors of the dots are used to distinguish the seven stem cell clusters. (D) Scaled expression levels (Z-score) of 8173 genes. The red color represents higher expression levels, and the blue color represents lower expression levels, respectively. Genes shown on the y-axis are hierarchically clustered. The color bar on the x-axis shows the stem cell clusters. (E) Expression levels (in log2 of TPM) of 41 histone genes corresponded with human orthologs. The darker orange color represents higher gene expression. Genes are arranged along the y-axis. Color bars along the x-axis represent the stem cell cluster with annotated factors.


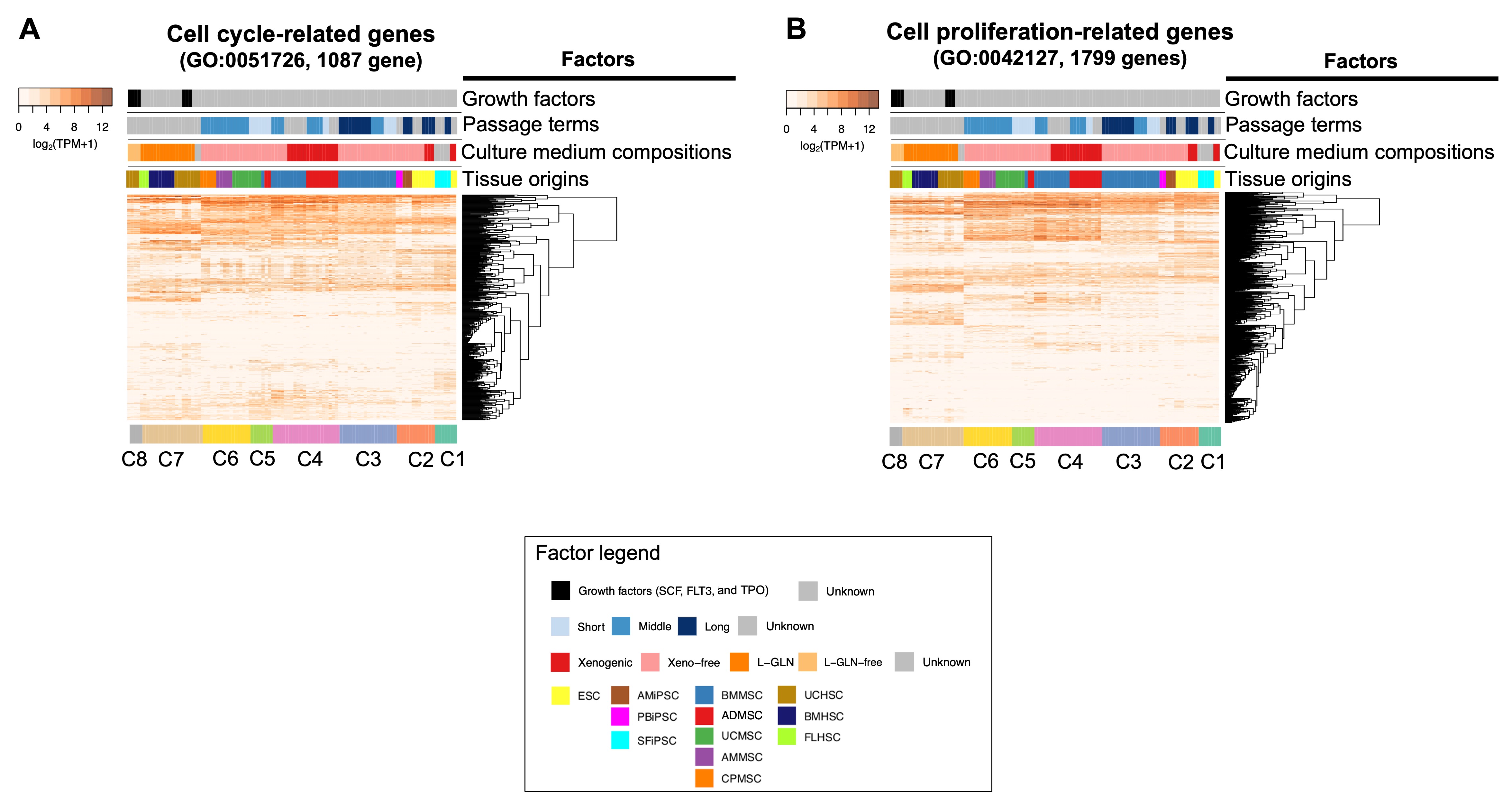


**Supplementary Figure S7.** Expression profiles of genes related to cell cycle and cell proliferation. Heatmap of expression profile for genes related to (A) cell cycle (GO:0051726, 1087 genes), (B) cell proliferation (GO:0042127, 1799 genes). The heatmaps show log2-fold transcription per million (TPM) of the genes. The darker orange color represents higher expression levels. Genes arranged along the y-axis are hierarchically clustered. Color bars in the x-axis represent four kinds of annotated factors (top) and the stem cell cluster (bottom), respectively.


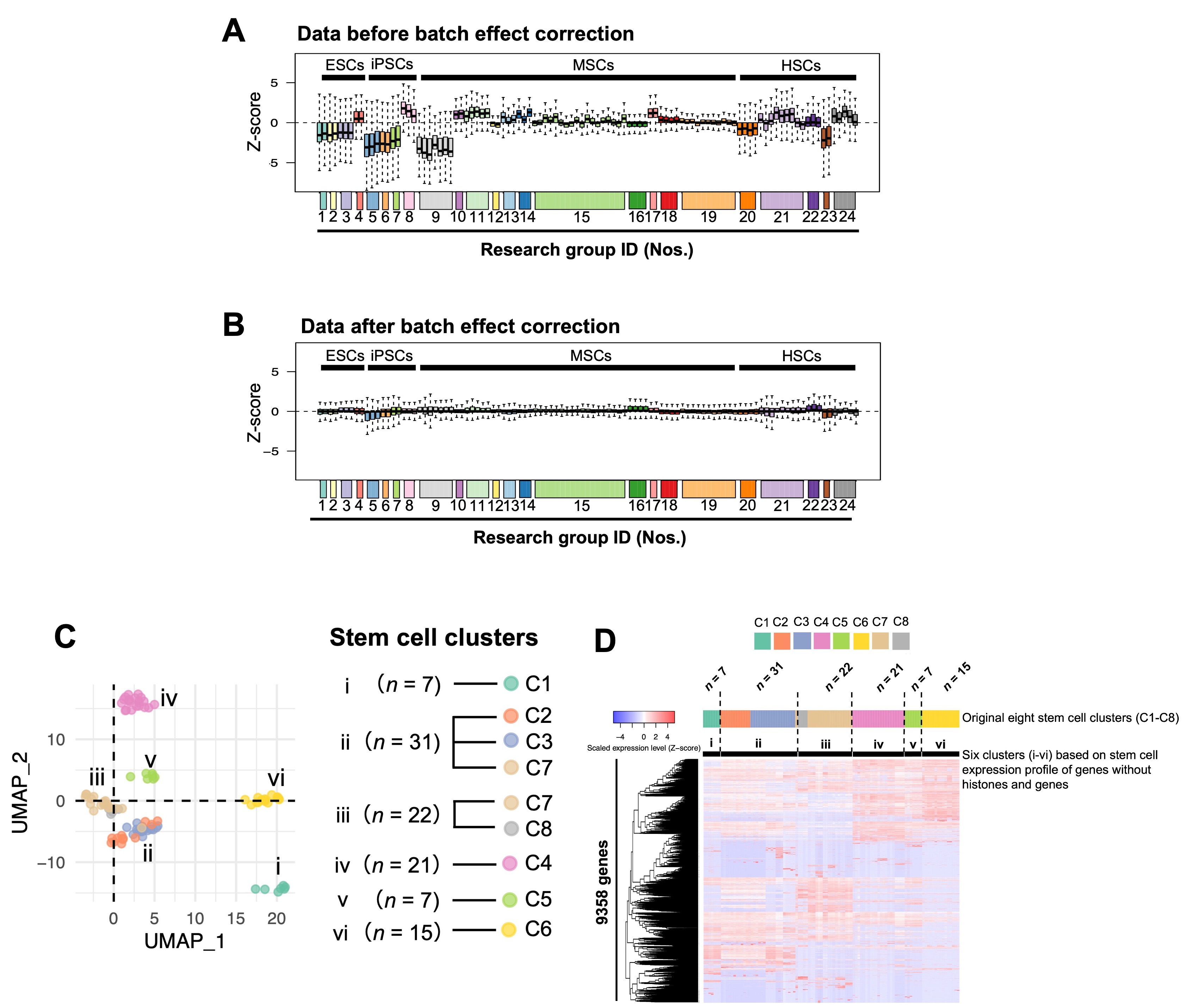


**Supplementary Figure S8.** Confirmation of the batch effect correction, UMAP, and stem cell clustering using genes without all histones and the top 100 genes highly correlated with the histones. (A, B) Relative gene expression levels (in Z-score) among RNA-seq data (A) before and (B) after the batch effect correction, respectively. The numbers in the horizontal axis show the research groups listed in Supplementary Table S1, and the different colors were used for their easy distinction. (C) Stem cell clustering. The stem cell cluster distribution (i-vi) was obtained using a K-nearest neighbor (KNN) graph, following dimensionality reduction by UMAP. An individual dot represents each stem cell sample. The colors of the dots correspond with clusters shown in Figure 2. (D) Heatmap of gene expression profiles among stem cells. Scaled expression levels (Z-score) of 9358 genes were investigated. The red color represents higher expression levels, and the blue color represents lower expression levels, respectively. Genes shown on the y-axis are hierarchically clustered. The color bar in the x-axis shows the two sets of stem cell classifications, eight clusters (C1-C8) and six clusters (i-vi).
